# Conditional knockout mice for the distal appendage protein CEP164 reveal its essential roles in airway multiciliated cell differentiation

**DOI:** 10.1371/journal.pgen.1007128

**Published:** 2017-12-15

**Authors:** Saul S. Siller, Himanshu Sharma, Shuai Li, June Yang, Yong Zhang, Michael J. Holtzman, Wipawee Winuthayanon, Holly Colognato, Bernadette C. Holdener, Feng-Qian Li, Ken-Ichi Takemaru

**Affiliations:** 1 Medical Scientist Training Program (MSTP), Stony Brook University, Stony Brook, New York, United States of America; 2 Graduate Program in Molecular and Cellular Pharmacology, Stony Brook University, Stony Brook, New York, United States of America; 3 Department of Pharmacological Sciences, Stony Brook University, Stony Brook, New York, United States of America; 4 School of Molecular Biosciences, College of Veterinary Medicine, Washington State University, Pullman, Washington, United States of America; 5 Division of Pulmonary and Critical Care Medicine, Department of Medicine, Washington University School of Medicine, St. Louis, Missouri, United States of America; 6 Department of Biochemistry and Cell Biology, Stony Brook University, Stony Brook, New York, United States of America; Washington University School of Medicine, UNITED STATES

## Abstract

Multiciliated cells of the airways, brain ventricles, and female reproductive tract provide the motive force for mucociliary clearance, cerebrospinal fluid circulation, and ovum transport. Despite their clear importance to human biology and health, the molecular mechanisms underlying multiciliated cell differentiation are poorly understood. Prior studies implicate the distal appendage/transition fiber protein CEP164 as a central regulator of primary ciliogenesis; however, its role in multiciliogenesis remains unknown. In this study, we have generated a novel conditional mouse model that lacks CEP164 in multiciliated tissues and the testis. These mice show a profound loss of airway, ependymal, and oviduct multicilia and develop hydrocephalus and male infertility. Using primary cultures of tracheal multiciliated cells as a model system, we found that CEP164 is critical for multiciliogenesis, at least in part, via its regulation of small vesicle recruitment, ciliary vesicle formation, and basal body docking. In addition, CEP164 is necessary for the proper recruitment of another distal appendage/transition fiber protein Chibby1 (Cby1) and its binding partners FAM92A and FAM92B to the ciliary base in multiciliated cells. In contrast to primary ciliogenesis, CEP164 is dispensable for the recruitment of intraflagellar transport (IFT) components to multicilia. Finally, we provide evidence that CEP164 differentially controls the ciliary targeting of membrane-associated proteins, including the small GTPases Rab8, Rab11, and Arl13b, in multiciliated cells. Altogether, our studies unravel unique requirements for CEP164 in primary versus multiciliogenesis and suggest that CEP164 modulates the selective transport of membrane vesicles and their cargoes into the ciliary compartment in multiciliated cells. Furthermore, our mouse model provides a useful tool to gain physiological insight into diseases associated with defective multicilia.

## Author summary

Lining the airways, brain ventricles, and oviducts, multicilia are small hair-like structures that beat in a whip-like motion to propel fluids, such as mucus, over cell surfaces. Dysfunction of multicilia arising from genetic perturbations is most prominently associated with a devastating disorder called primary ciliary dyskinesia (PCD). PCD is a rare genetic disease characterized by hydrocephalus, chronic airway infection, and infertility. Furthermore, defective airway multicilia have been implicated in several respiratory diseases, including cystic fibrosis, asthma, and chronic obstructive pulmonary disorder (COPD). While important to human health, the detailed molecular mechanisms of how multiciliated cells develop remain largely unknown. Here, we establish a new mouse model that lacks the key ciliary protein CEP164 in multiciliated cells. These mice recapitulate many symptoms of PCD patients such as hydrocephalus and infertility. We show that, in the absence of CEP164, differentiation of airway multiciliated cells is severely perturbed at multiple steps. Importantly, our data also suggest that CEP164 differentially regulates the proper recruitment of membrane-associated ciliary proteins. In summary, we have developed a powerful mouse model to study diseases affecting multicilia and shed light on novel roles of CEP164 in multiciliogenesis.

## Introduction

Cilia are evolutionarily conserved, microtubule-based organelles that project from the apical cell surface and perform a wide array of cellular functions [1–3]. Reflecting their diverse cellular tasks, many types of cilia exist, but they are generally categorized into two broad classes, primary and multicilia. Immotile primary cilia, which have a 9+0 microtubule arrangement, are present on most mammalian cell types, mediate signaling of multiple pathways including Hedgehog signaling, and sense the cellular environment [1]. Motile multicilia, on the other hand, have a 9+2 microtubule arrangement and are responsible for clearing mucus and debris from the airways, circulating cerebrospinal fluid in the brain ventricles, and providing the motive force for ovum transport along the oviduct (also called the fallopian tube) [4, 5]. Sperm flagella are also motile with a 9+2 axonemal structure. In recent years, the identification of human mutations in cilia-related genes, causative for a group of disorders known as ciliopathies, has highlighted the importance of primary cilia to human health and created great interest in the field [1–3]. On the other hand, multicilia have been linked to genetic disorders such as primary ciliary dyskinesia (PCD). Although there are exceptions, in most cases, PCD is caused by the immotility or abnormal motility of multicilia of normal length and number per cell [6, 7]. In addition, multicilia have been associated with several chronic respiratory disorders including chronic obstructive pulmonary disease (COPD) and asthma [6, 7]. These findings underscore the importance of elucidating the molecular mechanisms underlying the formation and function of multicilia.

Although primary and multicilia are thought to be produced through largely analogous pathways, differences exist [4, 5, 8]. Primary cilia are nucleated in a quiescent cell from the mother centriole, which is distinguished from the daughter centriole by the presence of the subdistal and distal appendages. The two centrioles surrounded by amorphous pericentriolar material constitute the centrosome. However, multiciliated cells must generate hundreds of centrioles through the direct duplication of existing centrioles and an acentriolar pathway via fibrogranular structures termed deuterosomes [4, 9]. After centrioles are formed in multiciliated cells, they mature through the acquisition of accessary structures, such as the subdistal and distal appendages [2, 10]. In both primary and multiciliogenesis, small vesicles then dock to the distal appendage and coalesce to form the larger ciliary vesicle [9, 11]. The ciliary vesicle is thought to promote docking of the centriole, now termed a basal body, to the apical cell surface by fusing with the apical cell membrane [12]. At this point, the axoneme extends from the basal body via the action of an intraciliary trafficking mechanism, called intraflagellar transport (IFT) [13].

The distal appendages, or transition fibers as referred to at the ciliary base, are nine radial fibrous extensions originating from the B-tubule at the distal end of the mother centriole or basal body [10]. A core unit composed of at least five proteins, CEP83/CCDC41, CEP89/CEP123, SCLT1, FBF1, and CEP164, has been reported [14]. Several functions have been ascribed to these proteins in primary ciliogenesis [15]; for example, FBF1 has been linked to IFT particle entry into the cilium while CEP83, CEP89, and CEP164 are critical for vesicle recruitment and ciliary vesicle biogenesis [16–19]. Rab small GTPases are known vesicle trafficking effectors and facilitate the assembly of ciliary vesicles and membranes at the distal appendage [10, 17, 20–22]. Specifically, during primary ciliogenesis, Rab11-positive vesicles are transported to the pericentrosomal region. Rabin8, a guanine nucleotide exchange factor (GEF) for Rab8, is then recruited by Rab11 to promote the local activation of Rab8, which in turn facilitates the efficient formation of ciliary vesicles and membranes. In addition to Rabs, ADP-ribosylation factor (Arf)/Arf-like (Arl) small GTPases also regulate primary ciliogenesis as well as targeting of ciliary proteins [23]. Interestingly, a recent report describes a novel role for the Eps15 homology domain (EHD) proteins EHD1 and EHD3 in ciliary vesicle formation in primary ciliogenesis [24]. Although these studies provide clear evidence that the distal appendage/transition fiber and its associated protein networks are necessary to build a primary cilium, little has been explored regarding their roles in multiciliogenesis.

We previously demonstrated that the 15-kDa coiled-coil protein Chibby1 (Cby1) localizes to the distal appendage/transition fiber and plays a key role in ciliogenesis [25–31]. Cby1-knockout (KO) mice display chronic sinusitis and otitis, polycystic kidneys, and sub-fertility as well as polydactyly and hydrocephalus at low frequency, due to defective primary and multicilia [25–27, 30, 31]. Recent studies in *Drosophila melanogaster* and *Xenopus laevis* highlight an evolutionarily conserved role for Cby1 in ciliogenesis [32, 33]. We further showed that CEP164, which is mutated in nephronophthisis and Bardet-Biedl syndrome (BBS), both of which are classified as ciliopathies [34, 35], directly interacts with and recruits Cby1 to the distal appendage/transition fiber of the mother centriole/basal body during primary ciliogenesis [25]. Cby1 then binds Rabin8 and facilitates an interaction between CEP164 and Rabin8. This leads to the recruitment and activation of Rab8 to promote the efficient assembly of ciliary vesicles and subsequent basal body docking to the apical plasma membrane. A crucial role for Cby1 in membrane association with and docking of basal bodies has been further demonstrated by studies in *D*. *melanogaster* [36]. Recently, we identified novel Cby1-interactors, the membrane-binding Bin/Amphiphysin/Rvs (BAR)-domain containing proteins, family with sequence similarity 92 members A and B (FAM92A and FAM92B) [37]. FAM92A and FAM92B are recruited to mother centrioles/basal bodies by Cby1 to facilitate ciliogenesis likely through regulation of membrane remodeling processes.

Centrosomal protein of 164 kDa (CEP164) was originally identified in a proteomic analysis of centrosomal proteins and a screen for modulators of ciliogenesis [38, 39]. CEP164-knockdown (KD) experiments in mammalian cultured cells revealed its functions in small vesicle docking to the distal appendage, at least in part, via the direct interactions between its C-terminal region and Rabin8 [17]. During primary ciliogenesis, the N-terminal WW motif of CEP164 has also been shown to bind and recruit Tau-tubulin kinase 2 (TTBK2) to the mother centriole [40, 41]. TTBK2 then phosphorylates the distal end-capping protein CP110 to promote the removal of CP110 from mother centrioles for the initiation of ciliogenesis. Thus, CEP164 is a key regulator of primary ciliogenesis; however, its role in multiciliogenesis remains largely unexplored.

Here, we report a novel conditional mouse model in which CEP164 is ablated from multiciliated cells. These mice show a severe reduction in the number of airway, ependymal, and oviduct multicilia, and ~20% die around weaning age with profound hydrocephalus. We found that CEP164 is important for proper multiciliogenesis by regulating ciliary vesicle formation and basal body docking. Experiments using primary cultures of mouse tracheal epithelial cells (MTECs) revealed that CEP164 is required for the normal basal body localization of Cby1 and its interactors FAM92A and FAM92B. Moreover, we provide evidence that CEP164 plays distinct roles in primary vs. multiciliogenesis and differentially controls the ciliary trafficking of membrane-associated proteins in multiciliated cells. Taken together, our study establishes a novel mouse model for multicilia-associated diseases and sheds light on the multiple indispensable roles of CEP164 in airway multiciliated cell differentiation.

## Results

### CEP164 is indispensable for early mouse embryogenesis

**Fig 1:**
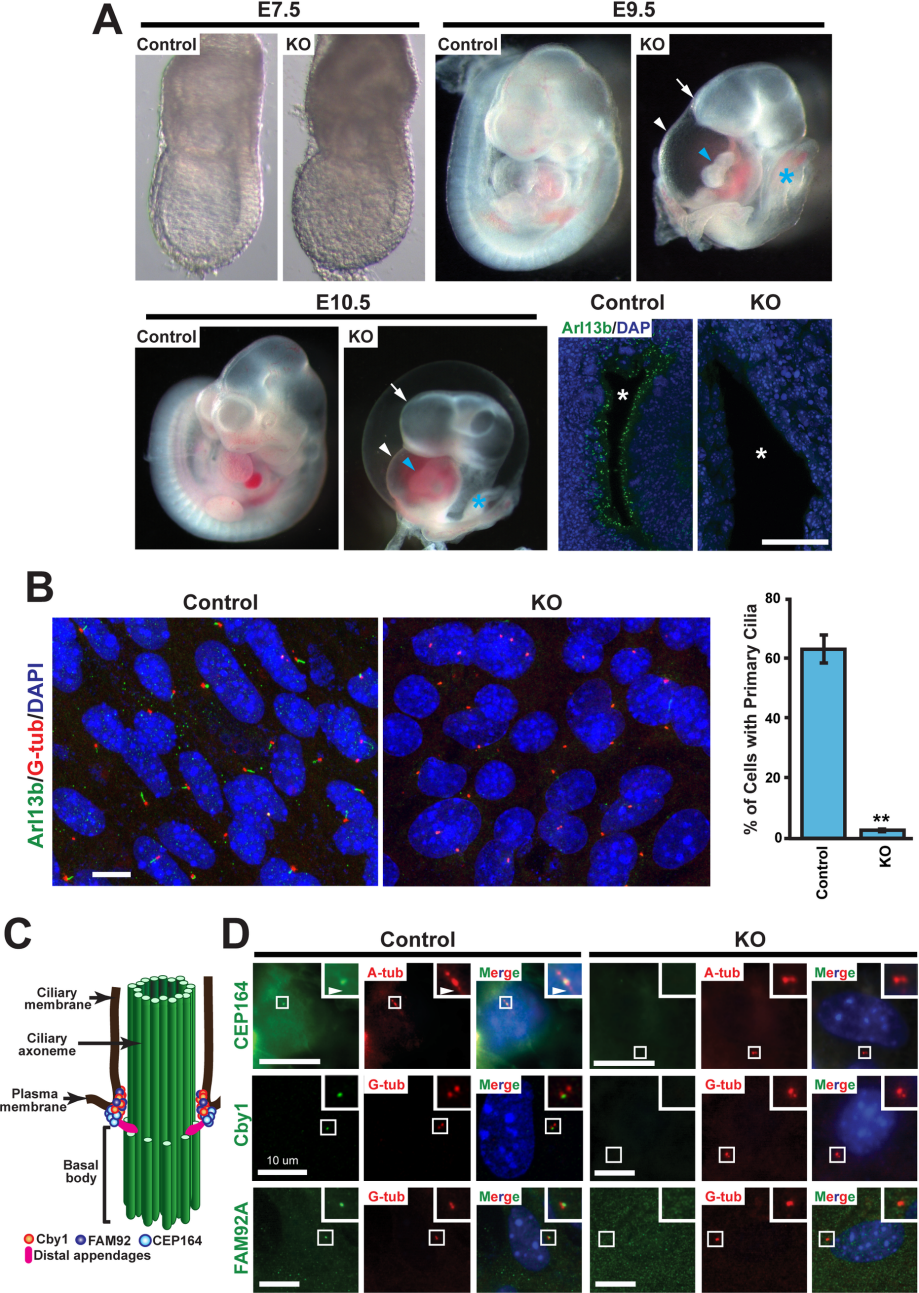
CEP164 is essential for early embryonic development and primary ciliogenesis. (A) Comparison of control (WT or heterozygous) embryos with CEP164-knockout (KO) littermates at E7.5, E9.5, and E10.5. At E7.5, KO embryos were indistinguishable from control littermates. In contrast, E9.5 and E10.5 KO embryos displayed holoprosencephaly (arrow), an edematous pericardial sac (white arrowhead), cardiac looping defects (blue arrowhead), and a truncated posterior trunk (blue asterisk). Confocal images of neural tube sections from E9.5 control and KO embryos are presented in lower right panels. Primary cilia were labeled with the ciliary marker Arl13b (green), and nuclei are visualized with DAPI staining (blue). Asterisks indicate the lumen of the neural tube. Scale bar, 35 μm. (B) Loss of primary cilia in CEP164-KO MEFs. Mouse embryonic fibroblasts (MEFs) were prepared from E8.5 CEP164-KO or control embryos, serum-starved for 48 hours to induce primary cilia, and immunostained for Arl13b (green) and the basal body marker γ-tubulin (G-tub) (red). Nuclei were stained with DAPI (blue). Scale bar, 10 μm. Quantification is shown on the right. >200 cells were counted for each of three independent MEF preparations per genotype. Error bars represent ±SEM. **, p<0.01. (C) Schematic depiction of the localization of Cby1, FAM92, and CEP164 at the ciliary base. Basal bodies and centrioles are barrel-shaped structures composed of nine microtubule triplets. During ciliogenesis, mother centrioles transform into basal bodies by acquiring accessary structures to assemble cilia. The axoneme is the detergent-insoluble cytoskeletal structure of the cilium including microtubules and their associated proteins. (D) Serum-starved MEFs were double-labeled for CEP164, Cby1, or FAM92A (green) and the ciliary marker acetylated α-tubulin (A-tub) or G-tub (red) as indicated. Nuclei were visualized by DAPI (blue). The boxed regions are enlarged in insets, highlighting the loss of CEP164, Cby1, and FAM92A centriolar localization in CEP164-KO MEFs. Arrowheads point to primary cilia. Scale bars, 10 μm.

CEP164 is composed of 1460 amino acids and contains a WW domain along with three coiled-coil domains (S1A Fig). To elucidate the physiological function of CEP164 in mammals, we obtained the CEP164 KO-first mouse line from the MRC-Harwell (S1B Fig) [42, 43]. This mouse line contains the promoter-driven Tm1a allele that carries *lacZ* gene and neomycin-resistance cassettes. As we initially expanded our CEP164 KO-first mouse colony, we noted that mice heterozygous for the KO-first allele appeared healthy and fertile while no homozygous mice were born, suggesting embryonic lethality. To address this possibility, we examined embryos from heterozygous intercrosses at various stages of gestation. At embryonic day (E) 7.5, CEP164-KO embryos showed no obvious morphological abnormalities; however, at E9.5 and E10.5, they exhibited holoprosencephaly, cardiac looping defects, an edematous pericardial sac, and a truncated posterior trunk (Fig 1A). These phenotypes are similar to those reported for mouse mutants for KIF3A and KIF3B [44–46], which are major components of the kinesin-II ciliary anterograde motor, providing further evidence for the essential role of CEP164 in primary ciliogenesis. Resorptions were consistently observed at E12.5 and all later stages examined. These data demonstrate that CEP164 is necessary for mammalian embryogenesis.

CEP164 has been shown to be essential for primary ciliogenesis in mammalian cultured cells and zebrafish embryos [17, 39, 40, 47, 48]. To determine whether CEP164 is necessary for primary ciliogenesis *in vivo*, we assessed the status of primary cilia in the neural tube of E9.5 CEP164-KO embryos using immunofluorescence (IF) staining for the ciliary marker Arl13b (Fig 1A). Primary cilia were abundant in the neural tube of control embryos but almost completely absent in that of CEP164-KO embryos. Consistent with this, mouse embryonic fibroblasts (MEFs) prepared from E8.5 CEP164-KO embryos showed a dramatic loss of primary cilia (2.7±0.3% ciliated KO MEFs vs. 62.3±4.1% ciliated control MEFs) (n>200 cells for each of three independent MEF preparations per genotype) (Fig 1B). These findings suggest that loss of primary cilia is, at least in part, responsible for the embryonic phenotypes observed.

We previously demonstrated that CEP164 physically interacts with Cby1 and is responsible for the recruitment of both Cby1 and FAM92A to the ciliary base to facilitate primary ciliogenesis in mammalian cultured cells using siRNA-mediated KD experiments (Fig 1C) [25, 37]. Indeed, in contrast to control MEFs, neither Cby1 nor FAM92A was detected at the centrioles of CEP164-KO MEFs (Fig 1D). We also confirmed the loss of CEP164 at the centrioles of CEP164-KO MEFs using IF staining (Fig 1D). Thus, our results validate previous data suggesting a fundamental role for CEP164 in the recruitment of Cby1 and FAM92A to basal bodies.

### Loss of CEP164 in FOXJ1-positive tissues in mice leads to severe deficits in multiciliogenesis and spermatogenesis

CEP164 plays an essential role in primary ciliogenesis; however, the role of CEP164 in multiciliogenesis had not been elucidated, and no CEP164-KO animal models were available to investigate its physiological functions *in vivo*. We therefore employed the CEP164 KO-first mouse line to generate a mouse model that lacks CEP164 in multiciliated cells (S1B Fig). To this end, a heterozygous CEP164 KO-first mouse was crossed with a flippase (Flp) deleter mouse to remove both the *lacZ* and neomycin-resistance cassettes. The resultant mouse (CEP164^fl/fl^) has two loxP sites flanking exon 4 of the *CEP164* gene, which encodes a part of the WW domain (S1A Fig). FOXJ1 is a forkhead transcription factor expressed in multiciliated cells in the airways, brain ventricles, and oviducts as well as in the testis [49, 50]. In airway multiciliated cells, FOXJ1 is expressed early during multiciliogenesis in ciliating cells that still possess a primary cilium and are initiating production of centrosomal proteins for centriole amplification [51]. Thus, we bred the CEP164^fl/fl^ mouse with a FOXJ1-Cre transgenic mouse that expresses Cre recombinase under the control of the FOXJ1 promoter [52]. Cre-mediated recombination results in the excision of exon 4 and a frameshift, leading to a truncation at amino acid position 65 (S1A Fig). Correct genotypes were verified by PCR (S1C and S1D Fig). A majority of FOXJ1-Cre;CEP164^fl/fl^ mice lived to adulthood without gross abnormalities, except for ~20% that succumbed to death due to severe hydrocephalus around weaning and another ~20% that exhibited mild hydrocephalus, which resolved itself later.

**Fig 2:**
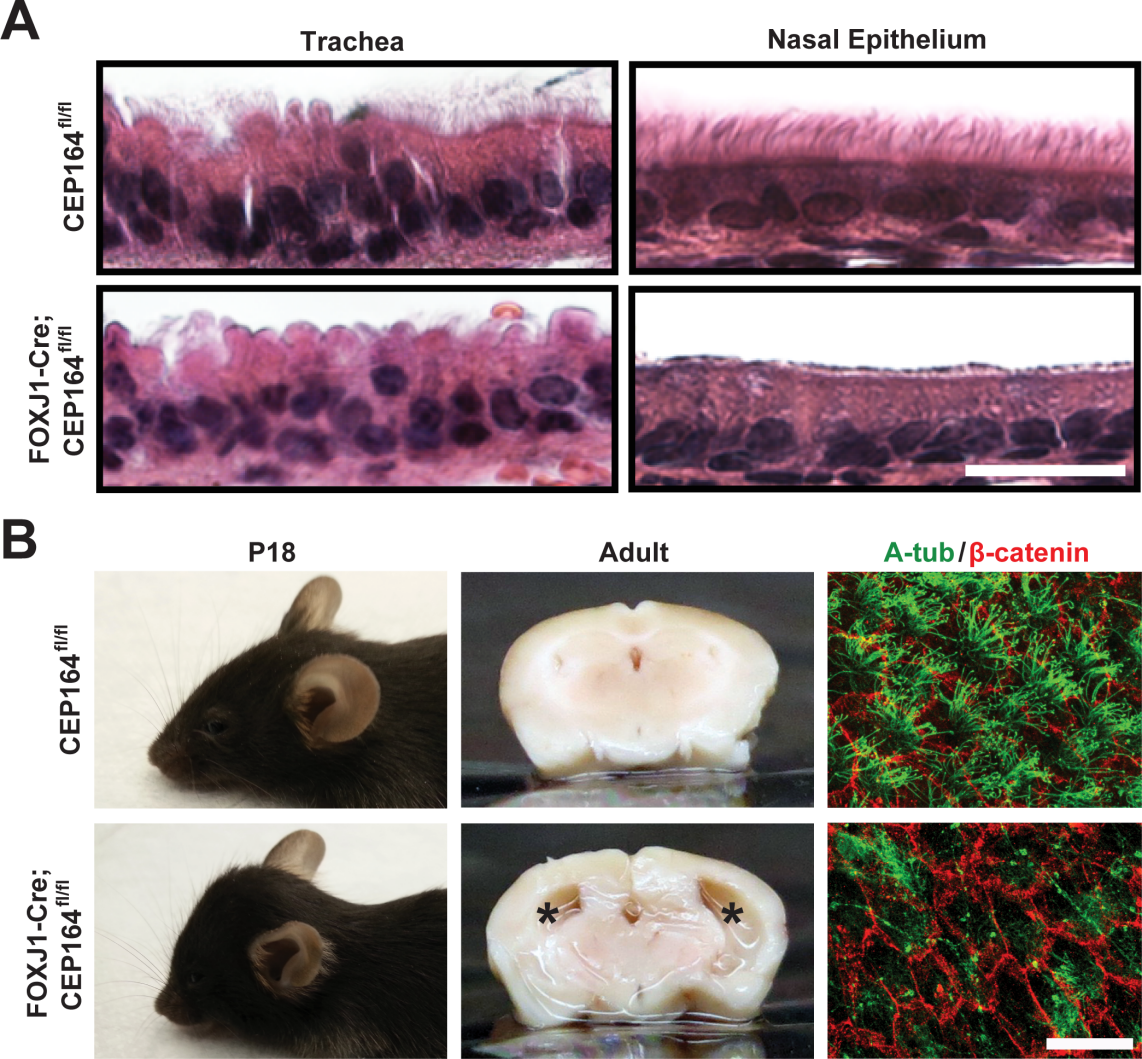
Ablation of CEP164 in the FOXJ1-positive tissues results in loss of airway and ependymal multicilia and hydrocephalus. (A) Hematoxylin and eosin (H&E)-stained tracheal and sinus sections from control CEP164^fl/fl^ and FOXJ1-Cre;CEP164^fl/fl^ adult mice. Scale bar, 20 μm. (B) Shown are lateral views of postnatal day 18 (P18) mice (left panels), coronal sections of adult brains (middle panels), and IF staining for A-tub (green) and β-catenin (red) in whole mounts of the adult subventricular zone (SVZ) (right panels). Asterisks denote enlarged lateral ventricular spaces indicative of hydrocephalus. Scale bar, 25 μm.

Histological assessment of the trachea and sinus (Fig 2A) from FOXJ1-Cre;CEP164^fl/fl^ adult mice showed a marked decrease in the number of airway multicilia in comparison to control specimens from CEP164^fl/fl^ mice. IF staining of tracheal sections for the ciliary marker acetylated α-tubulin (A-tub) showed significant loss of multicilia upon CEP164 deletion (S2 Fig). Indicative of impaired mucociliary clearance, these mice frequently produced coughing- or sneezing-like noises. As noted above, 19% of FOXJ1-Cre;CEP164^fl/fl^ mice displayed severe hydrocephalus with a prominently domed head around weaning (11 out of 58 mice) (Fig 2B, left panels); however, all FOXJ1-Cre;CEP164^fl/fl^ adult mice examined (n = 10) showed substantial ventricular enlargement (middle panels). The high penetrance of hydrocephalus prompted us to examine the status of ependymal multicilia by IF staining of whole mounts of the subventricular zone (SVZ). As expected, IF staining for A-tub demonstrated a clear reduction in the number of ependymal multicilia in FOXJ1-Cre;CEP164^fl/fl^ SVZ whole mounts compared to control CEP164^fl/fl^ samples (Fig 2B, right panels). Consistent with this, quantification of basal body patch area and displacement revealed significant perturbations in the organization of basal bodies at the apical surface of CEP164-KO ependymal multiciliated cells (S3 Fig).

**Fig 3:**
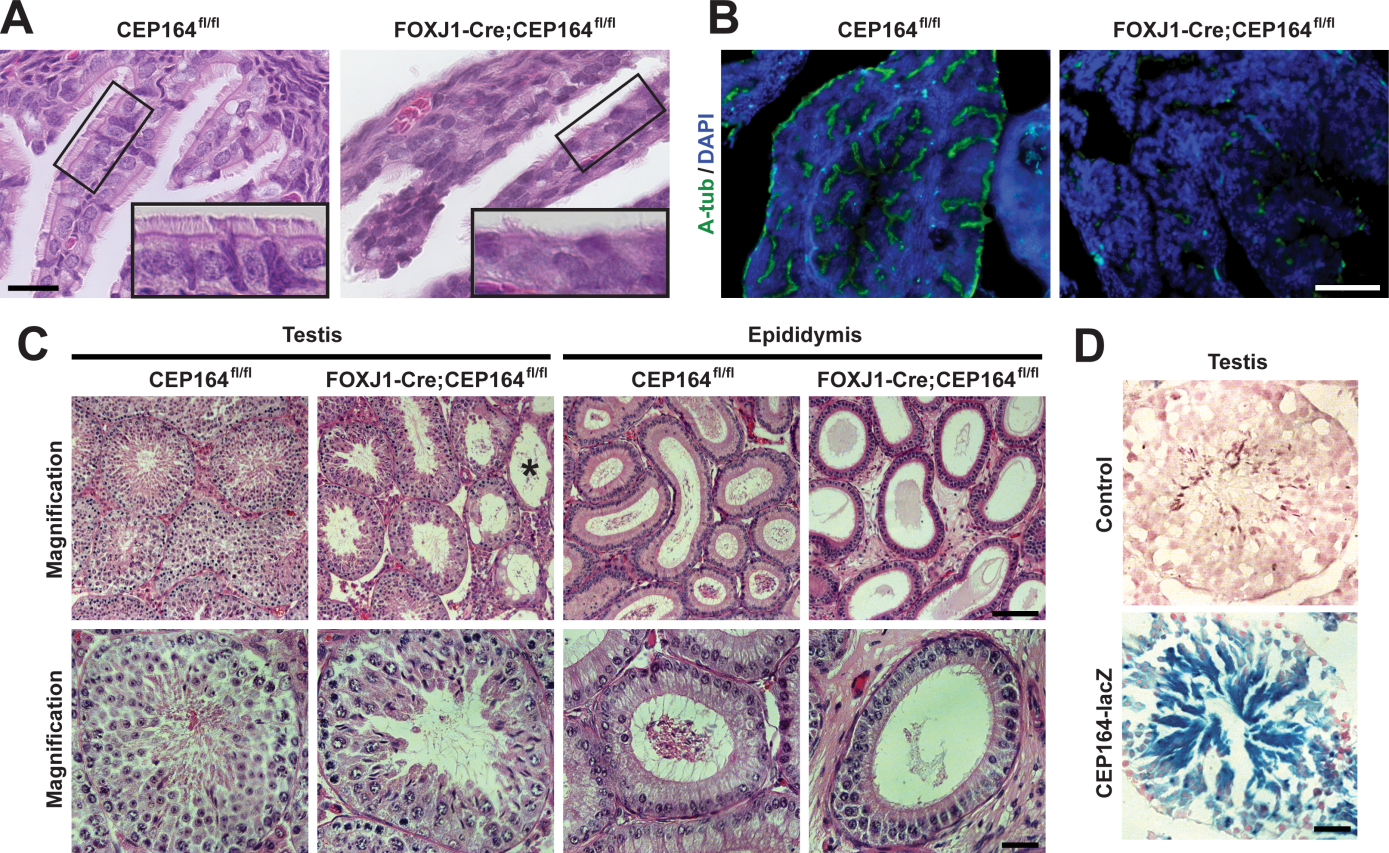
CEP164 plays an important role in proper development of female and male reproductive systems. (A) H&E staining of oviduct sections from CEP164^fl/fl^ and FOXJ1-Cre;CEP164^fl/fl^ adult mice. The boxed regions are enlarged in insets. Scale bar, 10 μm. (B) IF staining of oviduct sections for A-tub (green) and DAPI (blue). Scale bar, 50 μm. (C) H&E staining of both testis and epididymis from 3-month-old CEP164^fl/fl^ and FOXJ1-Cre;CEP164^fl/fl^ mice. Asterisk denotes seminiferous tubules lacking germ cells. Scale bars, 100 μm for low magnification and 40 μm for high magnification. (D) X-gal staining of testis sections from a control WT mouse and a mouse heterozygous for the CEP164 KO-first allele that contains a *lacZ* reporter. Scale bar, 40 μm.

We next examined reproductive tissues in FOXJ1-Cre;CEP164^fl/fl^ mice as FOXJ1 is highly expressed in the multiciliated cells of the oviduct epithelium as well as in the testis [49, 52]. In the oviduct of adult FOXJ1-Cre;CEP164^fl/fl^ mice, multicilia were reduced in number as evaluated by both histology (Fig 3A) and IF staining for A-tub (Fig 3B) compared to control CEP164^fl/fl^ tissues. However, FOXJ1-Cre;CEP164^fl/fl^ females were fertile, suggesting that the remaining multicilia are sufficient to sustain normal function. Alternatively, ciliary motility is not strictly required for female fertility. In stark contrast, FOXJ1-Cre;CEP164^fl/fl^ males were completely infertile. Histological analysis revealed variable degrees of degenerative changes in the seminiferous tubules of FOXJ1-Cre;CEP164^fl/fl^ adult testes. In general, we noticed a substantial reduction in the number of late-stage germ cells (Fig 3C). In a subset of seminiferous tubules, germ cells were entirely depleted with solely Sertoli cells present (Fig 3C, asterisk). Additionally, no mature sperm were detectable in the epididymis of FOXJ1-Cre;CEP164^fl/fl^ mice. In support of these extensive phenotypes, X-gal staining of testis sections from heterozygous CEP164 KO-first mice carrying a *lacZ* reporter showed broad CEP164 expression with particularly intense staining in differentiating spermatids (Fig 3D). Overall, these results demonstrate that FOXJ1-Cre;CEP164^fl/fl^ mice exhibit phenotypes consistent with impaired multi- and motile ciliogenesis and provide a useful model system to study the mechanisms of multiciliogenesis and its associated diseases.

### CEP164 is critical for ciliogenesis during differentiation of airway multiciliated cells

To gain insight into the molecular basis of defective multiciliogenesis in the absence of CEP164, we employed primary cultures of MTECs, a well-characterized *in vitro* model for airway differentiation and ciliogenesis [53]. MTEC cultures are created by seeding isolated tracheal epithelial cells at low density onto a semipermeable, collagen-coated membrane and permitting them to proliferate until confluent for ≤7 days. Differentiation then proceeds in a semi-synchronous manner after an air-liquid interface (ALI) is established with low-serum media. At 14 days post-ALI induction (ALId14), the cultures contain both multiciliated and non-ciliated cells and resemble the native tracheal epithelium.

Using the MTEC system, we first sought to determine the efficiency of Cre-mediated CEP164 removal as well as whether it has any impact on the multiciliated cell lineage. Hence, we performed IF staining of ALId14 MTECs for CEP164 and FOXJ1. While intense CEP164 signals were detectable at the ciliary base of FOXJ1-positive multiciliated cells in CEP164^fl/fl^ MTEC cultures at ALId14, CEP164 expression was lost or greatly reduced in ~90% of FOXJ1-positive multiciliated cells in FOXJ1-Cre;CEP164^fl/fl^ MTEC cultures (n>800 ciliated cells), revealing highly efficient Cre-mediated recombination (S4A Fig). On the other hand, it is possible that the efficiency of Cre recombination might vary among individual cells, leading to a partial/variable phenotype especially at early stages of multiciliogenesis. Interestingly, there was a modest decrease in the number of FOXJ1-positive cells in FOXJ1-Cre;CEP164^fl/fl^ (37.1±2.7%) vs. CEP164^fl/fl^ (47.6±3.0%) MTEC cultures (n>500 cells for each of three independent MTEC preparations per genotype) (S4B Fig). These data suggest that CEP164 may play some role in the maintenance and/or survival of multiciliated cells. Clearly, this requires further detailed investigation in the future.

**Fig 4:**
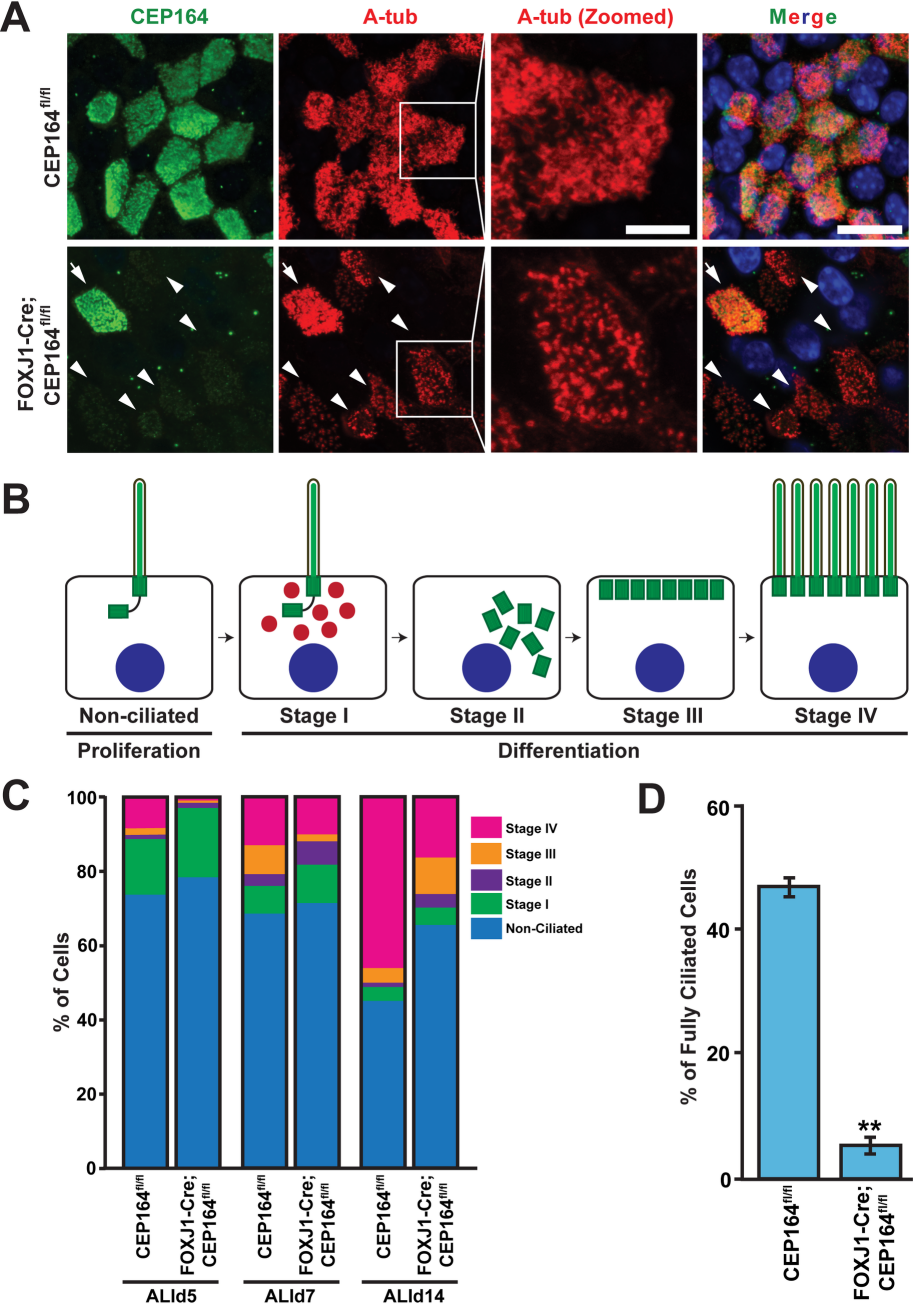
Ablation of CEP164 leads to defective airway multiciliogenesis. (A) ALId14 MTECs were stained for CEP164 (green) and A-tub (red). Nuclei were stained with DAPI (blue). Arrowheads denote CEP164-KO multiciliated cells with sparse, stubby cilia. Zoomed views of cilia are shown for the squared areas. Arrows indicate a multiciliated cell with CEP164 expression that escaped Cre-mediated recombination in FOXJ1-Cre;CEP164^fl/fl^ MTEC cultures. Scale bars, 10 μm and 5 μm for zoomed images. (B) Schematic model depicting the stages of multiciliated cell differentiation. See text for details. (C) Quantification of multiciliated cells at different stages (I-IV) of ciliogenesis. MTECs from CEP164^fl/fl^ and FOXJ1-Cre;CEP164^fl/fl^ mice were fixed at ALId5, d7, and d14 and immunostained for A-tub. n>225 total cells per ALI day from each of three independent MTEC preparations per genotype. (D) Quantification of fully ciliated cells. MTECs from CEP164^fl/fl^ and FOXJ1-Cre;CEP164^fl/fl^ mice were fixed at ALId14 and immunostained for A-tub. The percentages were calculated by dividing the number of fully ciliated cells with abundant cilia by total cell number. n>250 total cells from each of three independent MTEC preparations per genotype. Error bars represent ±SEM. **, p<0.01.

Next, we assessed the extent of multiciliogenesis in ALId14 MTECs from CEP164^fl/fl^ and FOXJ1-Cre;CEP164^fl/fl^ mice by IF staining for CEP164 and A-tub (Fig 4A). As expected, CEP164-KO multiciliated cells showed profound defects in ciliogenesis. However, we noticed that CEP164-KO multiciliated cells were able to extend cilia, albeit short and sparse (Fig 4A, zoomed image), in contrast to the absolute requirement for CEP164 in primary ciliogenesis [17, 39]. Four different stages of centriole formation and ciliogenesis in multiciliated cells are defined: Stage I, appearance of centrosomal protein foci; Stage II, centriole replication; Stage III, centriole dispersion and migration; Stage IV, axonemal elongation (Fig 4B) [8]. To precisely quantify the percentages of multiciliated cells at each stage, we fixed CEP164^fl/fl^ and FOXJ1-Cre;CEP164^fl/fl^ MTECs at ALId5, d7, and d14 and conducted IF staining for A-tub. As shown in Fig 4C, impaired multiciliogenesis in FOXJ1-Cre;CEP164^fl/fl^ MTEC cultures was evident at ALId5 and more pronounced at ALId14 with a large decrease in the number of stage IV multiciliated cells and concomitant increases in the numbers of early stage multiciliated cells (n>225 total cells for each ALI day from each of three independent MTEC preparations per genotype). The increased number of non-ciliated cells at ALId14 was in line with the decreased number of FOXJ1-positive cells in ALId14 FOXJ1-Cre;CEP164^fl/fl^ MTECs (S4B Fig). Moreover, a vast majority of the stage IV multiciliated cells in FOXJ1-Cre;CEP164^fl/fl^ MTEC cultures extended only short and scarce cilia at ALId14 (Fig 4D). Corroborating this observation, CEP164^fl/fl^ MTEC cultures had 46.5±1.4% of total cells that were fully ciliated with abundant cilia, whereas only 4.9±1.1% of cells in FOXJ1-Cre;CEP164^fl/fl^ MTEC cultures appeared fully ciliated (n>250 total cells from each of three MTEC independent preparations per genotype), which most likely corresponds to CEP164-positive multiciliated cells that escaped Cre-mediated recombination. Collectively, these data indicate that loss of CEP164 in airway multiciliated cells results in defective ciliogenesis and multiciliated cell differentiation.

### CEP164 is required for ciliary vesicle formation and basal body docking during airway multiciliated cell differentiation

**Fig 5:**
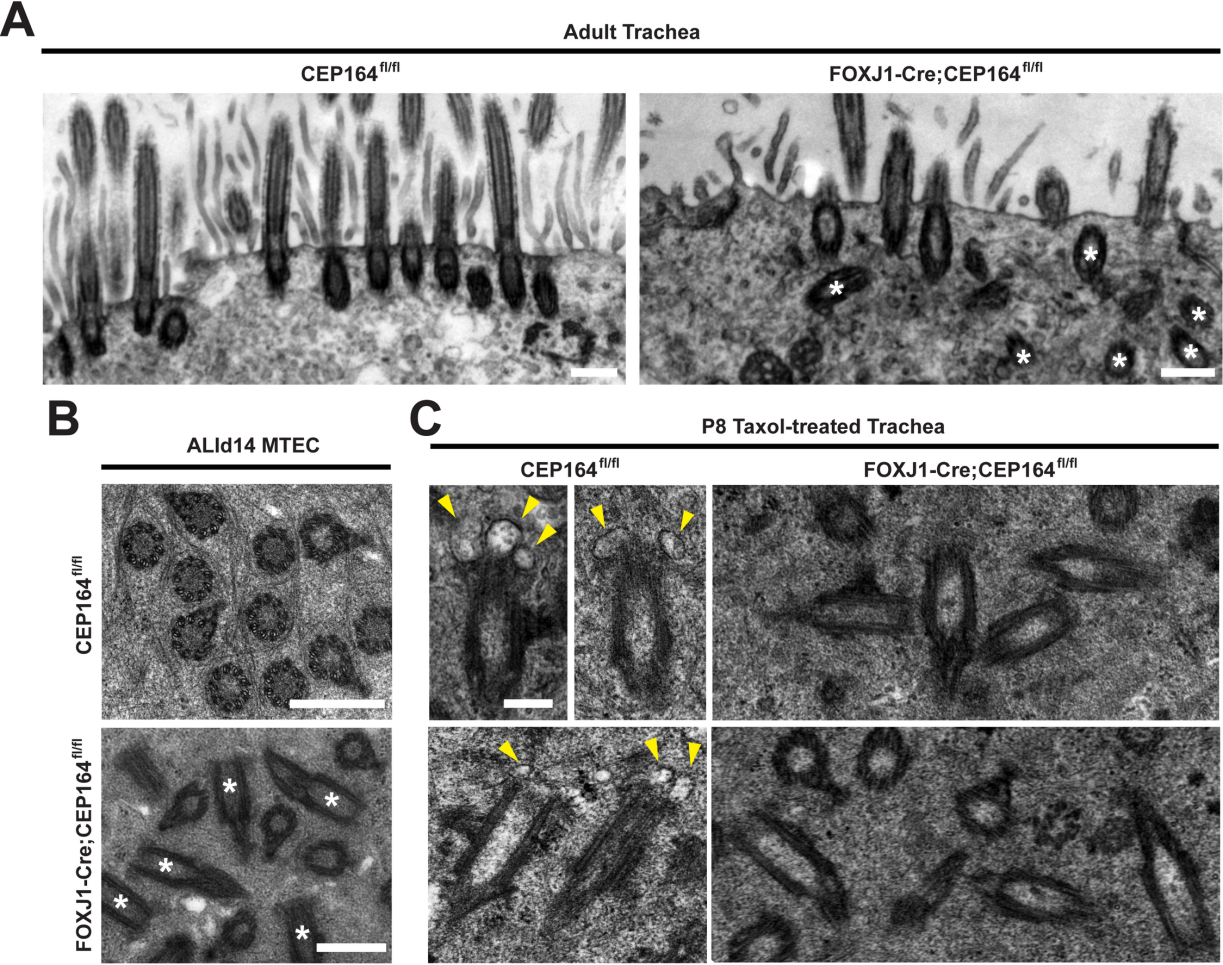
CEP164 regulates small vesicle recruitment to basal bodies in multiciliated cells. (A) TEM images of multiciliated cells from CEP164^fl/fl^ and FOXJ1-Cre;CEP164^fl/fl^ adult tracheas. Asterisks depict multiple cytoplasmic basal bodies in FOXJ1-Cre;CEP164^fl/fl^ trachea. Scale bar, 500 nm. (B) TEM images of cross sections through the apical region of multiciliated cells from ALId14 MTECs derived from CEP164^fl/fl^ and FOXJ1-Cre;CEP164^fl/fl^ adult tracheas. Asterisks depict multiple cytoplasmic, misoriented basal bodies in FOXJ1-Cre;CEP164^fl/fl^ multiciliated cells. Scale bars, 500 nm. (C) TEM images of CEP164^fl/fl^ and FOXJ1-Cre;CEP164^fl/fl^ P8 tracheas subjected to *ex vivo* culture in the presence of taxol to enrich vesicle-bound basal bodies. Arrowheads denote vesicles attached to the distal end of the cytoplasmic centriole in control CEP164^fl/fl^ samples. Scale bar, 200 nm.

During primary ciliogenesis, CEP164 plays a pivotal role in recruitment of small vesicles to the distal appendages of mother centrioles for assembly of ciliary vesicles [17]. To examine whether CEP164 similarly regulates vesicle recruitment and subsequent basal body docking during multiciliogenesis, we performed transmission electron microscopy (TEM) on both CEP164^fl/fl^ and FOXJ1-Cre;CEP164^fl/fl^ adult tracheas. In control CEP164^fl/fl^ multiciliated cells, 98% of basal bodies were properly docked to the apical cell surface with cilia extending into the lumen (n = 167 basal bodies from 12 ciliated cells) (Fig 5A). In contrast, 48–83% of basal bodies were found undocked in the cytoplasm of FOXJ1-Cre;CEP164^fl/fl^ multiciliated cells with only a few cilia (n = 176 basal bodies from 10 ciliated cells). In agreement with the IF staining results of MTECs (Fig 4A), we frequently noted shortened cilia in FOXJ1-Cre;CEP164^fl/fl^ adult tracheas (S5B Fig). We also confirmed the presence of many undocked, cytoplasmic basal bodies in ALId14 MTECs from FOXJ1-Cre;CEP164^fl/fl^ mice using TEM (Fig 5B). Furthermore, we found that the transition fibers as well as the Y-linkers of the transition zone were present in the absence of CEP164 (S5A, S5C and S5D Fig), suggesting that CEP164 is not an essential structural component of the transition fibers and does not influence transition zone ultrastructure.

Basal body docking defects often result from the inability of distal appendages to recruit small vesicles in order to assemble ciliary vesicles [10, 15]. CEP164 has been shown to be responsible for the recruitment of small vesicles to distal appendages during early stages of primary ciliogenesis in human retinal pigment epithelial (RPE1) cells [17]. To test if this is the case in multiciliated cells, P8 tracheas were cultured *ex vivo* in the presence of the microtubule-stabilizing agent taxol and subjected to TEM analysis. Taxol has previously been shown to block apical migration of basal bodies and enrich for basal bodies bound to vesicles in multiciliated cells [54]. As shown in Fig 5C, in control CEP164^fl/fl^ tracheas, 68% of cytoplasmic basal bodies were associated with vesicles, whereas only 35% of basal bodies in FOXJ1-Cre;CEP164^fl/fl^ tracheas were attached to vesicles (n = 68 and 81 basal bodies for CEP164^fl/fl^ and FOXJ1-Cre;CEP164^fl/fl^, respectively, from three tracheas per genotype). Of note, without 3D reconstruction, these numbers do not precisely represent the actual number of centrioles associated with vesicles, but rather the numbers detected on thin TEM sections. Collectively, our TEM data support the notion that CEP164 plays key roles in small vesicle recruitment and ciliary vesicle formation during multiciliogenesis.

### CEP164 recruits Chibby1, FAM92A, and FAM92B to basal bodies in multiciliated cells

**Fig 6:**
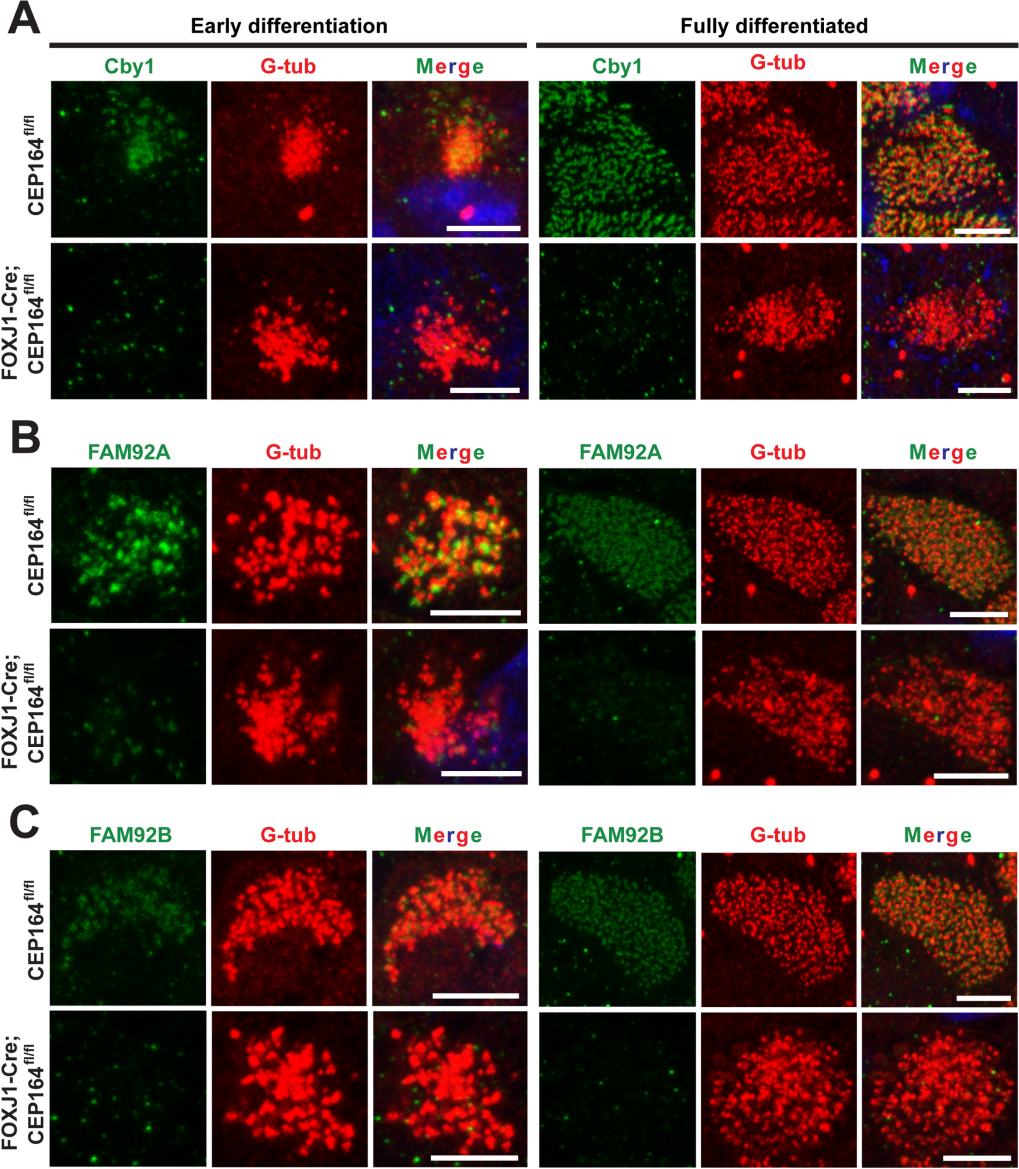
CEP164 recruits Cby1 and FAM92 proteins to basal bodies in multiciliated cells. MTECs from CEP164^fl/fl^ and FOXJ1-Cre;CEP164^fl/fl^ mice were immunostained for Cby1 (A), FAM92A (B), or FAM92B (C) (green) and G-tub (red) as indicated. DAPI staining (blue) marks nuclei in merged images. Scale bars, 5 μm.

We previously reported that Cby1 is important for ciliary vesicle formation and basal body docking in airway multiciliated cells [25]. We also demonstrated that, during primary ciliogenesis, CEP164 is essential for recruitment of Cby1 to the distal appendages of mother centrioles via protein-protein interactions. Cby1 then recruits the BAR domain-containing proteins FAM92A and FAM92B to basal bodies to facilitate primary ciliogenesis [37]. IF staining of MTECs revealed that Cby1 recruitment to basal bodies was lost or substantially reduced at both early and fully differentiated stages (Fig 6A). At ALId14, ~65% of CEP164-KO multiciliated cells showed diminished recruitment of Cby1 to basal bodies (n = 300 ciliated cells from three independent MTEC preparations). Similarly, the basal body recruitment of FAM92A and FAM92B was clearly diminished in 50–65% of CEP164-KO multiciliated cells (n>250 ciliated cells at ALId14 for each protein from three independent MTEC preparations) (Fig 6B and 6C). These data indicate that CEP164 lies upstream of Cby1, FAM92A, and FAM92B and recruits them to the distal appendages/transition fibers to promote ciliary vesicle formation, basal body docking, and multiciliated cell differentiation.

### The loss of CEP164 in multiciliated cells does not overtly influence the basal body localization of IFT components and CP110

**Fig 7:**
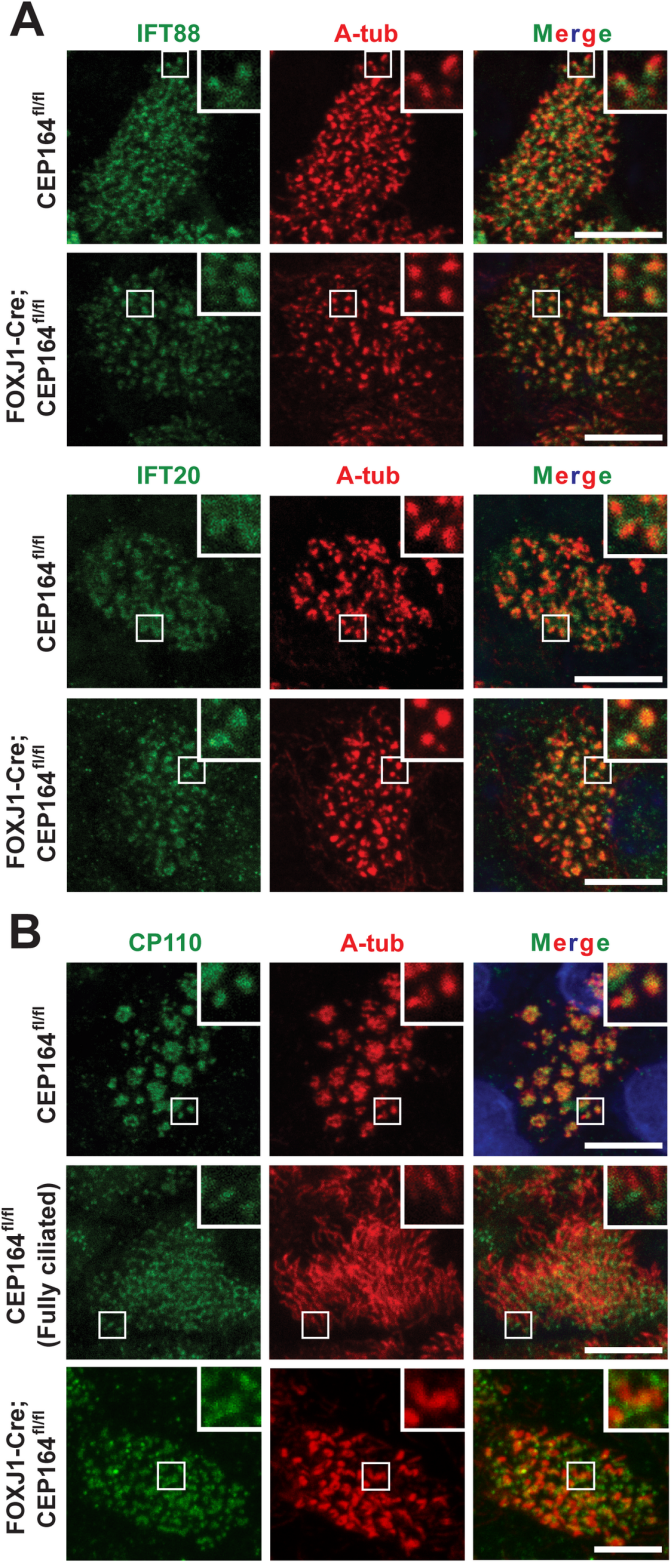
CEP164 is dispensable for the basal body localization of IFT88, IFT20, and CP110 in multiciliated cells. ALId14 MTECs from CEP164^fl/fl^ and FOXJ1-Cre;CEP164^fl/fl^ mice were immunostained for IFT components (A) or CP110 (B) (green) and A-tub (red). Nuclei were detected with DAPI (blue). All multiciliated cells were at early stage IV except for the fully differentiated cell (Fully Ciliated) imaged to show the clear basal body localization of CP110. The insets show zoomed views of the squared areas. Scale bars, 5 μm.

It was demonstrated that CEP164 KD leads to a significant reduction in the levels of IFT components at the base of primary cilia in RPE1 cells [17, 40]. We therefore examined the effects of CEP164 loss on the localization of the IFT components IFT88 and IFT20 in multiciliated cells (Fig 7A). Surprisingly, in contrast to primary cilia, both IFT proteins were clearly detectable at basal bodies in CEP164-KO multiciliated cells at similar levels to control multiciliated cells. During primary ciliogenesis, CEP164 recruits TTBK2 to mother centrioles [40, 41]. TTBK2 in turn promotes removal of the distal end-capping protein CP110 and recruitment of IFT proteins to initiate ciliogenesis. Thus, CEP164-KD RPE1 cells fail to remove CP110 from the mother centriole, thereby preventing ciliogenesis from proceeding upon serum starvation [40, 41]. In contrast, we found that, in multiciliated cells, CP110 was constitutively present at nascent centrioles as well as at the basal bodies of elongating and mature cilia (Fig 7B). The basal body localization of CP110 was not overtly affected in CEP164-KO multiciliated cells. TTBK2 was weakly detectable at the ciliary base and more brightly at the tip of a subset of cilia at comparable levels in both control and CEP164-KO multiciliated cells (S6A Fig). These findings suggest that CEP164 is dispensable for the proper localization of IFT particles, CP110, and TTBK2 to centrioles/basal bodies in multiciliated cells and highlight potential differences between primary and multiciliogenesis.

### Distribution of ciliary membrane proteins is perturbed in CEP164-KO multiciliated cells

**Fig 8:**
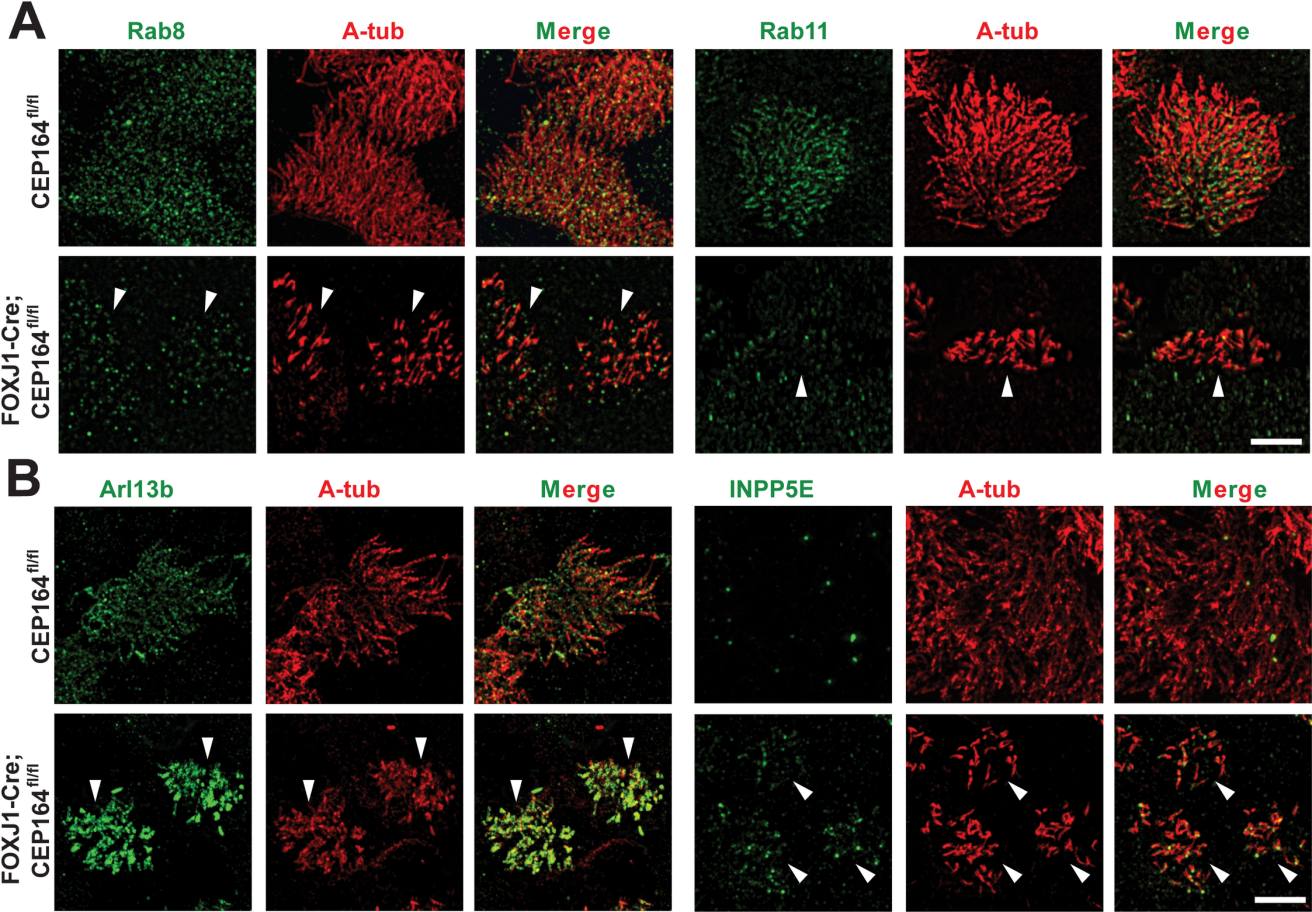
CEP164 is required for the proper targeting of ciliary membrane proteins in multiciliated cells. (A) CEP164^fl/fl^ or FOXJ1-Cre;CEP164^fl/fl^ MTECs at ALId14 were immunostained for Rab8 or Rab11 (green) and A-tub (red) and subjected to super-resolution SIM imaging. Arrowheads point to individual CEP164-KO multiciliated cells. Scale bar, 5 μm. (B) ALId14 MTECs from CEP164^fl/fl^ and FOXJ1-Cre;CEP164^fl/fl^ mice were immunostained for Arl13b or INPP5E (green) and A-tub (red) and imaged by SIM. Arrowheads indicate individual CEP164-KO multiciliated cells. Scale bar, 5 μm.

During primary ciliogenesis, the small GTPase Rab11 recruits Rab8 GEF Rabin8, which in turn recruits and activates Rab8 at centrosomes [20–22]. Rab8 then promotes membrane trafficking to the base of cilia to facilitate ciliary membrane assembly. CEP164 is known to bind Rabin8 and mediates Rab8 recruitment and activation [17]. Furthermore, Cby1 binds CEP164 to facilitate the CEP164-Rabin8 interaction and Rab8 activation, thereby promoting ciliary vesicle formation and subsequent basal body docking during airway multiciliated cell differentiation [25]. We therefore hypothesized that CEP164 might affect the Rab11-Rab8 cascade in multiciliated cells and immunostained ALId14 MTECs from CEP164^fl/fl^ and FOXJ1-Cre;CEP164^fl/fl^ mice with antibodies for Rab8 and Rab11 (Fig 8A). Utilizing super-resolution structured illumination microscopy (SIM), we found that the ciliary and basal body localization of both Rab8 and Rab11 was substantially reduced in CEP164-KO compared to control multiciliated cells. Of particular note, Rab11 has been reported to predominantly localize to a pericentrosomal compartment in cycling cells or a peri-basal body region in quiescent cells with primary cilia [17, 20, 22]. In contrast, Rab11 localization extended to a proximal region of multicilia, again highlighting differences between primary and multicilia. These data point to potential alterations in the trafficking and formation of ciliary membranes in CEP164-KO multiciliated cells.

Next, we sought to determine if other ciliary membrane proteins exhibit altered localization patterns upon CEP164 loss. The ciliary protein ADP-ribosylation factor-like 13b (Arl13b) is a small GTPase that specifically associates with the ciliary membrane via palmitoylation and functions in vesicle and ciliary trafficking as well as multiple other cellular processes [55–57]. Additionally, Arl13b forms a functional complex with CEP164 to target the lipid phosphatase inositol polyphosphate-5-phosphatase E (INPP5E) to the primary cilium [58]. INPP5E is a prenylated protein important for primary ciliogenesis and maintenance of proper ciliary membrane lipid composition [59–61]. Furthermore, genetic mutations in Arl13b and INPP5E are linked to the ciliopathy Joubert syndrome [62, 63]. Hence, we investigated whether the loss of CEP164 has any effect on the ciliary localization of Arl13b and INPP5E. Surprisingly, SIM imaging revealed that, in CEP164-KO multiciliated cells, Arl13b robustly accumulated in the short cilia and that the ciliary localization of INPP5E was modestly, yet consistently, increased along the entire length of the short cilia (Fig 8B). The ciliary accumulation of Arl13b was also observed at early ciliation phases in ALId5 MTEC cultures, although that of INPP5E was not clearly detectable (S6B Fig). In CEP164-KO MEFs, weak to moderate signals for Arl13b and INPP5E were consistently observed at centrioles (S7 Fig). These results, combined with the diminished ciliary recruitment of Rabs in CEP164-KO multiciliated cells, imply that CEP164 might be involved in the trafficking and formation of ciliary membranes in multiciliated cells.

## Discussion

In spite of a large portion of the population affected by genetic and/or chronic disorders involving multicilia, only a few surviving mouse models exist to interrogate the mechanisms of their formation and physiology. Here, we report a viable mouse model that lacks the distal appendage/transition fiber protein CEP164 in FOXJ1-positive cells of the airways, brain, oviduct, and testis (S1 Fig). We demonstrated that CEP164 removal in these tissues results in a profound loss of multicilia in the airway, ependymal, and oviduct epithelia as well as development of hydrocephalus and male infertility (Figs 2–4). Therefore, our FOXJ1-Cre;CEP164^fl/fl^ mouse model provides a powerful tool to study diseases of multicilia, such as PCD, and to further elucidate how defective multicilia contribute to the pathology of chronic respiratory diseases, such as cystic fibrosis, asthma, and COPD.

Cilia play essential roles in various aspects of embryonic development such as tissue patterning and organogenesis [1, 64]. CEP164-KO embryos exhibit holoprosencephaly, an edematous cardiac sac, heart looping defects, and a truncated posterior trunk at E9.5–10.5 (Fig 1A), leading to embryonic lethality. Intriguingly, these phenotypes resemble those of the mouse mutants for KIF3A [44, 45] and KIF3B [46], which are components of the plus end-directed kinesin-II microtubule motor that carries IFT particles and cargoes to the tip of cilia. These similarities in phenotype indicate that CEP164 may play an essential role in primary ciliogenesis during early embryogenesis. In agreement with this notion, we found that CEP164-KO MEFs fail to develop primary cilia (Fig 1B).

Our findings also revealed a critical role for CEP164 in male reproductive development (Fig 3C and 3D). Interestingly, mature sperm were not present in the epididymis of FOXJ1-Cre;CEP164^fl/fl^ mice. In severe cases, germ cells were completely depleted, suggesting that CEP164 may have a fundamental function in spermatogonial stem cells. Besides sperm flagella, germ cells in mammalian testes lack primary cilia [65, 66]. Thus, CEP164 may play cilia-independent roles during spermatogenesis. Future studies are clearly warranted to address the biological functions of CEP164 in the testis.

In line with its function during primary ciliogenesis, our results suggest that CEP164 is critical for the recruitment of small vesicles to the distal appendages of centrioles and subsequent assembly of ciliary vesicles during multiciliogenesis. Furthermore, CEP164 is required for the proper basal body localization of the downstream effectors Cby1, FAM92A, and FAM92B in airway multiciliated cells (Fig 6). At present, we cannot rule out the possibility that, besides its role in basal body docking, CEP164 may play an additional role in other aspects of multiciliogenesis such as centriole amplification and cilium elongation. Our work also highlights differences in the requirements for CEP164 in primary vs. multiciliogenesis. While CEP164 has been shown to be necessary for IFT88 recruitment to basal bodies in primary cilia [17], both IFT88 and IFT20 localize to the ciliary base in CEP164-KO multiciliated cells (Fig 7A). Additionally, CEP164 physically interacts with TTBK2 to promote the removal of the centriolar distal end-capping protein CP110 to initiate primary ciliogenesis [40]. In contrast, CP110 is clearly present at the basal bodies of mature cilia in airway multiciliated cells in a CEP164-independent manner (Fig 7B). Consistent with these findings, a recent report, using *Xenopus* epidermal multiciliated cells, demonstrated that CP110 localizes to basal bodies and may have unique functions in basal body apical transport and ciliary adhesion complex formation during multiciliogenesis [67]. Hence, it will be of importance to determine shared and distinct mechanisms between primary vs. multiciliogenesis. This may contribute to the development of targeted therapies for symptoms associated with defective primary vs. muticilia.

Interestingly, we found that the ciliary localization of membrane-associated proteins is perturbed in CEP164-KO multiciliated cells. We observed a significant reduction in the levels of ciliary Rab8 and Rab11 (Fig 8A). Previously, CEP164 has been shown to interact with Rab8 GEF Rabin8 and recruit Rab8 to primary cilia [17]. Our data support this model during multiciliogenesis. Distinct from primary cilia where Rab11 is detectable at pericentrosomal regions [20, 22], Rab11 localization extends into the proximal portion of multicilia. It is possible that Rab11 is also present in primary cilia at very low levels beyond the detection limit of fluorescence microscopy. Alternatively, Rab11 may have additional unique functions in multiciliogenesis. In contrast to the Rabs, we observed increases in the ciliary localization of Arl13b and INPP5E in CEP164-KO multiciliated cells (Fig 8B). While we cannot completely exclude the possibility that the increased ciliary localization of Arl13b and INPP5E in the absence of CEP164 results from the recruitment of all the protein to a few remaining immature cilia, these findings are still surprising in light of a prior report that CEP164 forms a multiprotein complex with Arl13b and INPP5E and is important for their trafficking to primary cilia [58]. Based on these data, we propose that, in multiciliated cells, CEP164 functions in the selective transport of certain vesicle types carrying unique cargos into the cilium. In doing so, CEP164 recruits Rab-positive membrane vesicles and limits the proportion of Arl13b- and INPP5E-containing vesicles. This model also concurs with the notion that the transition fibers act as a ciliary gate that regulates the entry and exit of ciliary proteins and vesicles [10, 15]. FBF1, another distal appendage/transition fiber protein, has been shown to facilitate the entry of IFT particles into the cilium [19]. Therefore, CEP164 may function in an analogous manner to FBF1 for ciliary membrane vesicles and ciliary membrane proteins.

In summary, our data support a crucial role for CEP164 in multiciliogenesis. CEP164 recruits Cby1, FAM92A, and FAM92B along with the Rab11-Rab8 axis to basal bodies to facilitate ciliary vesicle formation and subsequent basal body docking. During cilium elongation and maintenance, CEP164 may play a role in selective transport of certain types of vesicle with distinct cargos to the ciliary compartment. Finally, our CEP164 conditional KO mouse model will provide a basis for future investigations into the molecular mechanisms of primary and multiciliogenesis *in vivo* as well as the pathogenesis and mechanisms of ciliopathies.

## Materials and methods

### Ethics statement

All mice were handled in accordance with NIH guidelines, and all protocols were approved by the Institutional Animal Care and Use Committee (IACUC) of Stony Brook University (#2010–1393).

### Generation of FOXJ1-Cre;CEP164^fl/fl^ mice

CEP164 KO-first mice, which contain the promoter-driven Tm1a allele, were obtained from the MRC-Harwell, which distributes these mice on behalf of the European Mouse Mutant Archive [42, 43]. CEP164 KO-first mice were crossed with the Flp deleter mouse line B6(C3)-Tg(Pgk1-FLPo)10Sykr/J (The Jackson Laboratory, #011065) to generate CEP164^fl/fl^ mice [68]. Removal of the *lacZ* and neomycin-resistance cassettes was confirmed by polymerase chain reaction (PCR) genotyping analysis and subsequent electrophoresis. Subsequently, CEP164^fl/fl^ mice were crossed with FOXJ1-Cre mice [52] to generate FOXJ1-Cre;CEP164^fl/fl^ mice lacking CEP164 in multiciliated cells and the testis. A colony of CEP164 KO-first mice was maintained by intercrossing heterozygous mice while FOXJ1-Cre;CEP164^fl/fl^ mice were generated by breeding FOXJ1-Cre;CEP164^fl/+^ with CEP164^fl/fl^ mice. Primers for genotyping were: WT allele for CEP164 KO-first, 5’-CCATCTGTCCAGTACCATTAAAAA-3’ and 5’-CCCAGAATACAACATGGGAGA-3’ (215 bp); KO allele for CEP164 KO-first, 5’-CCATCTGTCCAGTACCATTAAAAA-3’ and 5’-GAACTTCGGAATAGGAACTTCG-3’ (148 bp); CEP164 floxed allele, 5’-CCATCTGTCCAGTACCATTAAAAA-3’ and 5’-CCCAGAATACAACATGGGAGA-3’ (WT allele, 215 bp; floxed allele, 415 bp).

### Histology and X-gal staining

Trachea, testis, and oviduct from adult mice were fixed with 4% paraformaldehyde (PFA) in phosphate-buffered saline (PBS), pH 7.4, overnight at 4°C, paraffin-embedded, sectioned at 5 μm, stained with hematoxylin and eosin using standard protocols, and mounted with Permount (Fischer Scientific). For X-gal staining, testes from control WT or heterozygous CEP164 KO-first mice were fixed with 2% PFA and 0.25% glutaraldehyde in PBS overnight at 4°C, embedded in Optimal Cutting Temperature (OCT) compound (Fisher Scientific), and snap-frozen in liquid nitrogen-cooled 2-methylbutane. The tissues were then sectioned at 5 μm, washed twice for 5 minutes each in wash buffer (0.01% sodium deoxycholate, 2 mM MgCl_2_, and 0.02% NP-40 in PBS), incubated with X-gal (1 mg/ml) in wash buffer for 48 hours at room temperature, washed twice for 5 minutes in wash buffer, and mounted with Permount.

### Primary cultures of MEFs and MTECs

MEFs were prepared from E8.5 embryos of intercrosses between heterozygous CEP164 KO-first mice as previously described [28, 69], and extra-embryonic tissue was used for genotyping analysis. In brief, embryos were placed in 0.05% trypsin-EDTA, minced, and incubated in 0.05% trypsin-EDTA for 20 minutes at 37°C. Dissociated cells were plated out on glass coverslips in a 48-well plate and cultured in Dulbecco’s Minimum Essential Medium (DMEM) supplemented with 10% FBS (Invitrogen) and 100 U/ml penicillin/streptomycin. MEF cultures were allowed to grow until confluent, at which point ciliogenesis was induced by serum starvation for 48 hours.

MTECs were isolated and cultured as previously described [25, 28, 53]. Briefly, tracheas were dissected from 2- to 6-month-old CEP164^fl/fl^ and FOXJ1-Cre;CEP164^fl/fl^ mice (typically 4 tracheas per genotype per preparation), and tracheal epithelial cells were harvested after overnight incubation with 1.5 mg/ml pronase (Roche) at 4°C. Isolated MTECs were seeded onto collagen-coated Transwell permeable membranes made of either polycarbonate or polyester (6.5-mm diameter and 0.4-μm pore size; Corning Costar). Cultures were then allowed to proliferate in MTEC Plus media with retinoic acid (RA) until confluent, at which time an ALI was established and 2% NuSerum media with RA was provided only in the basal chamber of the Transwell (ALId0). MTECs were cultured until ALId14 to ensure full differentiation, unless otherwise noted.

### Immunofluorescence staining

IF staining was achieved using standard protocols as previously described [25, 28]. Briefly, MEF coverslips and MTEC membranes were fixed in either 4% PFA in PBS or ice-cold methanol-acetone (1:1) for 20 minutes at 4°C, washed three times with PBS for 10 minutes at 4°C, and blocked for 1 hour at room temperature with antibody diluent (5% bovine serum albumin [BSA] and 0.2% Triton X-100 in PBS) and 5% goat serum. MEF samples were incubated with primary and secondary antibodies for 1 hour each at room temperature. MTEC membranes were incubated with primary antibody overnight at 4°C, followed by 1 hour of blocking with 5% goat serum in antibody diluent prior to secondary antibody incubation for 1 hour at room temperature. Subsequently, samples were washed three times with PBS for 5 minutes each. Finally, DAPI counterstain was performed for 2 minutes at room temperature, followed by two 5-minute PBS washes. Specimens were then mounted with Fluoromount-G (SouthernBiotech). For analysis of primary cilia in the neural tube, E9.5 embryos were fixed in 4% PFA, cryoprotected with 30% sucrose and embedded in OCT compound for sectioning, followed by the IF staining procedure as described above. For IF staining of oviducts, paraffin sections were subjected to antigen retrieval with citrate buffer (pH 6.0), blocked with normal horse serum, and incubated with primary and secondary antibodies, followed by mounting with Prolong Gold with DAPI (Invitrogen). For primary antibody information, see S1 Table. The secondary antibodies used were: goat anti-rabbit IgG conjugated with either DyLight 488 or DyLight 549 and horse anti-mouse IgG conjugated with either DyLight 488 or DyLight 549 (Vector Laboratories).

### SVZ whole mount dissection and analysis

SVZ whole mounts were dissected as described previously [70]. Briefly, adult mice were anesthetized and decapitated. After brain removal, the lateral wall of the lateral ventricle was dissected and fixed in 4% PFA in PBS for 30 minutes on ice. Whole mounts were washed with PBS, blocked in blocking solution (10% donkey serum with 0.1% Triton X-100 in PBS), and incubated with primary antibodies for 24 hours at 4°C and secondary antibodies for 2 hours at room temperature in blocking solution. The secondary antibodies used were: goat anti-mouse IgG1 conjugated with DyLight 549 and goat anti-mouse IgG2b conjugated with DyLight 549 (Jackson ImmunoResearch). Whole mount fields were randomly selected for imaging from the anterior-dorsal region of the SVZ.

Images were processed and quantified using the FIJI/ImageJ software as previously described [70]. Outlines of the apical borders of ependymal multiciliated cells and the borders of basal body patches were traced manually in FIJI/ImageJ. Absolute areas were directly calculated and reported whereas fractional areas were calculated by dividing the basal body patch area by the apical cell surface area. The centroid of each area was calculated in FIJI/ImageJ, and the vector from the center of the cell and center of the basal body patch was then calculated based on those values. Basal body patch displacement was calculated by taking the magnitude of this vector. Fractional displacement was calculated by dividing the magnitude of the vector running from the center of the cell to the center of the basal body patch by the magnitude of a manually drawn vector running from the center of the cell through the center of the basal body and terminating at the cell border.

### Fluorescence imaging

Epifluorescence images were taken on a Leica DMI6000B epifluorescence microscope with an HCX PL Fluotar 100X/1.3 NA oil objective equipped with a DFC300FX camera. Confocal images were acquired from either a Leica SP5 or SP8X confocal microscope with a HC PL APO 100X/1.4 NA oil objective. For SIM imaging, MTECs were imaged using a Nikon N-SIM with a 100x/1.49 NA oil objective equipped with an Andor iXon3 897 EMCCD camera. All confocal and epifluorescence images were analyzed with Leica Application Suite X software while SIM images were analyzed with Nikon NIS-Elements image analysis software. Finally, all images were further processed with Adobe Photoshop and Illustrator.

### Transmission electron microscopy

Samples used for TEM were processed using standard techniques [25, 31]. Briefly, MTEC membranes and adult tracheas were fixed by immersion in 2.5% PFA and 2% glutaraldehyde in PBS overnight at 4°C. After fixation, samples were washed in PBS, placed in 2% osmium tetroxide in PBS, dehydrated in a graded series of ethanol, and embedded in Embed812 resin (Electron Microscopy Sciences). Ultrathin sections of 80 nm were cut with a Leica EM UC7 ultramicrotome and placed on Formvar-coated slot copper grids. Sections were then counterstained with uranyl acetate and lead citrate and viewed with a FEI Tecnai12 BioTwinG^2^ electron microscope. Digital images were acquired with an XR-60 CCD digital camera system (Advanced Microscopy Techniques).

### Tracheal culture and quantification of centrioles bound to vesicles

Centrioles in tracheal multiciliated cells were analyzed for the presence or absence of docked vesicles as previously described [25]. In brief, tracheas were dissected from P8 mice and cultured for 16 hours in a 5% CO_2_ atmosphere at 37°C in DMEM media supplemented with 10% FBS, 100 U/ml penicillin/streptomycin, 1 μg/ml insulin (Sigma-Aldrich), and 300 ng/ml dexamethasone (Sigma-Aldrich) in the presence of 1 μM paclitaxel (Sigma-Aldrich). Tracheas were then processed for TEM as described above.

### Statistical analysis

Two-tailed Student’s t-tests were used for quantification analysis as indicated, and p<0.05 was considered significant. In the figures, asterisks indicate p-values as follows: *, p<0.05; and **, p<0.01.

## Supporting information

S1 Fig Generation of FOXJ1-Cre;CEP164^fl/fl^ mice. (A) Schematic diagram of CEP164 protein structure illustrating the WW domain and the three coiled-coiled (CC) domains. The N-terminal portion of the protein encoded by exon 4 (ex4), which was removed upon Cre-mediated recombination, is depicted. The numbers indicate amino acid positions. (B) Shown are the original CEP164 KO-first allele, the floxed (fl) allele after removal of *lacZ* and neomycin cassettes upon crossing with flippase (Flp) deleter mice, and the final allele with exon 4 excised after Cre-mediated recombination driven by the FOXJ1 promoter. (C) PCR genotyping analysis confirming the generation of the CEP164^fl/fl^ mouse. The locations for genotyping primers (P1 and P2) for detection of the floxed allele (415 bp) and wild-type (WT) allele (215 bp) are indicated by green arrows in (B). (D) PCR genotyping analysis using tail genomic DNA confirming the generation of the FOXJ1-Cre;CEP164^fl/fl^ mouse.
(TIF)

S2 Fig Deletion of CEP164 in multiciliated tissues leads to significant loss of multicilia in the trachea. Tracheal sections from CEP164^fl/fl^ and FOXJ1-Cre;CEP164^fl/fl^ adult mice were immunostained for A-tub (green). Nuclei were detected with DAPI. Scale bar, 100 μm.
(TIF)

S3 Fig CEP164 is important for ependymal multiciliated cell maturation. (A) SVZ whole mount preparations from CEP164^fl/fl^ or FOXJ1-Cre;CEP164^fl/fl^ adult mice were immunostained for G-tub (white) and β-catenin (red). β-Catenin demarcates the cell boundaries, and γ-tubulin labels basal bodies that are found in patches in ependymal multiciliated cells. Scale bar, 25 μm. (B) Quantification of basal body patch areas. Basal body patch areas relative to total apical cell surface areas are significantly reduced in CEP164-KO ependymal multiciliated cells. (C) Quantification of displacement of basal body patches. The displacement of the basal body patches from the cell center relative to the radius of the apical cell surface is significantly increased in the absence of CEP164. For all quantification, n = 3. Error bars represent ±SEM. *, p<0.05; **, p<0.01.
(TIF)

S4 Fig Efficient removal of CEP164 by FOXJ1-Cre-mediated recombination in multiciliated cells in MTEC cultures. (A) MTECs were prepared from CEP164^fl/fl^ and FOXJ1-Cre;CEP164^fl/fl^ mice, fixed at ALId14, and immunostained for FOXJ1 (green) and CEP164 (red). Nuclei were stained using DAPI (blue). ~90% of multiciliated cells in MTEC cultures from FOXJ1-Cre;CEP164^fl/fl^ mice lost CEP164 expression. Scale bar, 25 μm. (B) Quantification of FOXJ1-positive multiciliated cells. The percentage of FOXJ1-positive cells in FOXJ1-Cre;CEP164^fl/fl^ MTECs was moderately reduced (~10%) in comparison to CEP164^fl/fl^ MTECs. >500 cells were counted for each of three independent MTEC preparations per genotype. Error bars represent ±SEM. **, p<0.01.
(TIF)

S5 Fig Transmission electron microscopy reveals short cilia as well as intact transition fibers and transition zone structures in CEP164-KO multiciliated cells. (A) Structure of multicilia. CP, cilia proper; BP, basal plate; TZ, transition zone, BB, basal body; TF, transition fiber (arrowheads). Scale bar, 100 nm. (B) Elongated cilia were abundant in cross-sections of tracheas from CEP164^fl/fl^ adult mice while short cilia were frequently found in tracheas from FOXJ1;CEP164^fl/fl^ adult mice. Scale bar, 500 nm. (C) Nine transition fibers from the microtubule triplets of the basal body were present in cross-sections of multicilia in ALId14 MTEC cultures from both CEP164^fl/fl^ and FOXJ1-Cre;CEP164^fl/fl^ mice. Scale bars, 100 nm. (D) Y-linkers within the transition zone were visible in cross-sections of multicilia in ALId14 MTEC cultures from both CEP164^fl/fl^ and FOXJ1-Cre;CEP164^fl/fl^ mice. Scale bars, 100 nm.
(TIF)

S6 Fig Effects of CEP164 deletion on the ciliary localization of TTBK2 and Arl13b in multiciliated cells. (A) ALId14 MTECs from CEP164^fl/fl^ and FOXJ1-Cre;CEP164^fl/fl^ mice were immunostained for TTBK2 (green) and the ciliary/basal body maker A-tub (red). Nuclei were detected with DAPI (blue). (B) ALId5 MTECs were immunostained for Arl13b (green) and A-tub (red) as indicated. Multiciliated cells at early ciliation phases are shown. Scale bars, 10 μm.
(TIF)

S7 Fig Effects of loss of CEP164 on the ciliary localization of Arl13b and INPP5E in MEFs. Mouse embryonic fibroblasts (MEFs) were prepared from E8.5 CEP164-KO or control embryos and serum-starved for 48 hours to induce primary cilia. MEFs were double-labeled for Arl13b or INPP5E (green) and the ciliary marker acetylated α-tubulin (A-tub). Nuclei were visualized by DAPI (blue). The boxed regions are enlarged in insets. Scale bar, 10 μm.
(TIF)

S1 Table Primary antibodies used for IF staining. (TIF)
