# Supplementary figures and images for: Conditional knockout mice for the distal appendage protein CEP164 reveal its essential roles in airway multiciliated cell differentiation
Source: PLoS Genet. 2017 Dec 15;13(12):e1007128. doi: 10.1371/journal.pgen.1007128 (PMC5747467; doi:10.1371/journal.pgen.1007128)

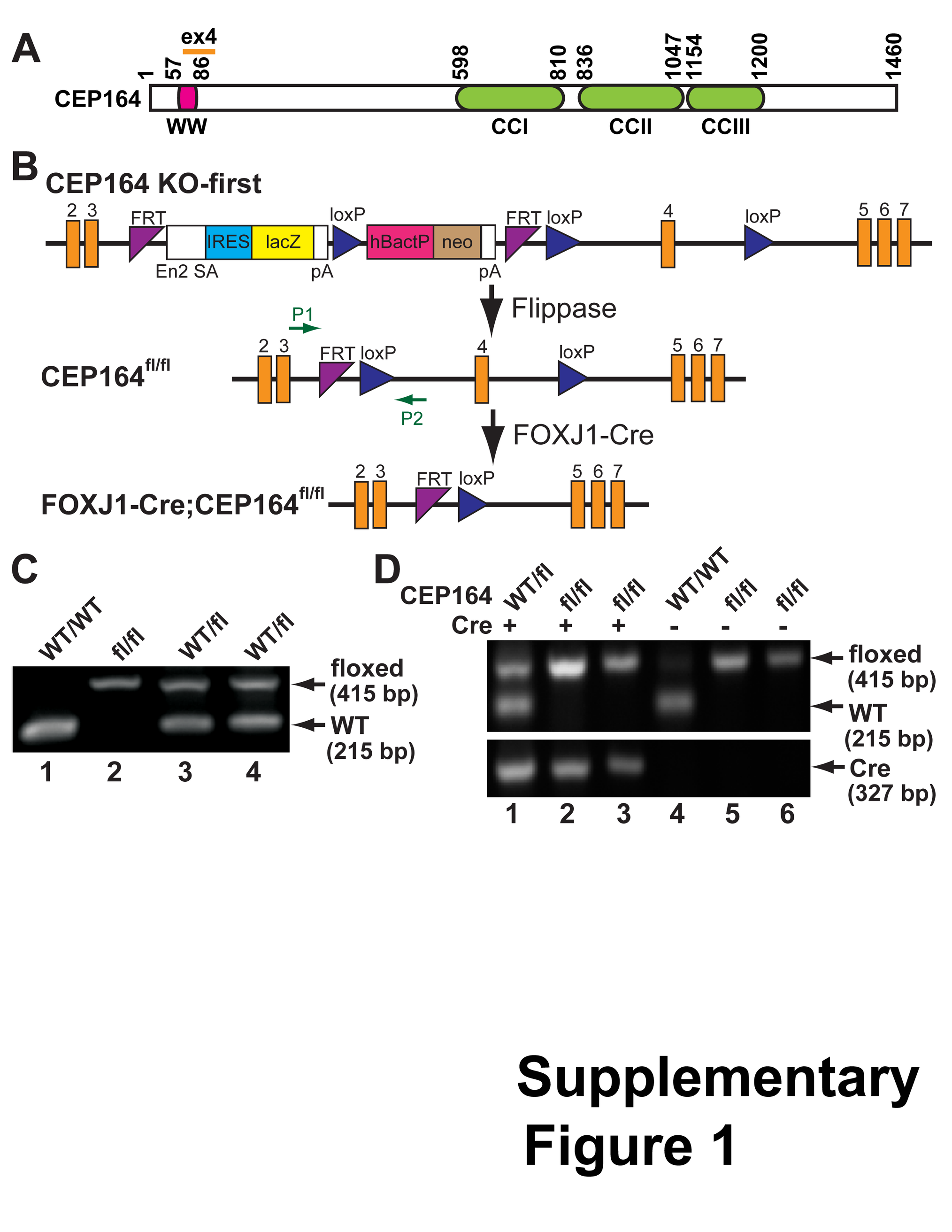

Supplement: S1 Fig — (A) Schematic diagram of CEP164 protein structure illustrating the WW domain and the three coiled-coiled (CC) domains. The N-terminal portion of the protein encoded by exon 4 (ex4), which was removed upon Cre-mediated recombination, is depicted. The numbers indicate amino acid positions. (B) Shown are the original CEP164 KO-first allele, the floxed (fl) allele after removal of lacZ and neomycin cassettes upon crossing with flippase (Flp) deleter mice, and the final allele with exon 4 excised after Cre-mediated recombination driven by the FOXJ1 promoter. (C) PCR genotyping analysis confirming the generation of the CEP164fl/fl mouse. The locations for genotyping primers (P1 and P2) for detection of the floxed allele (415 bp) and wild-type (WT) allele (215 bp) are indicated by green arrows in (B). (D) PCR genotyping analysis using tail genomic DNA confirming the generation of the FOXJ1-Cre;CEP164fl/fl mouse. (TIF) [file pgen.1007128.s001.tif]

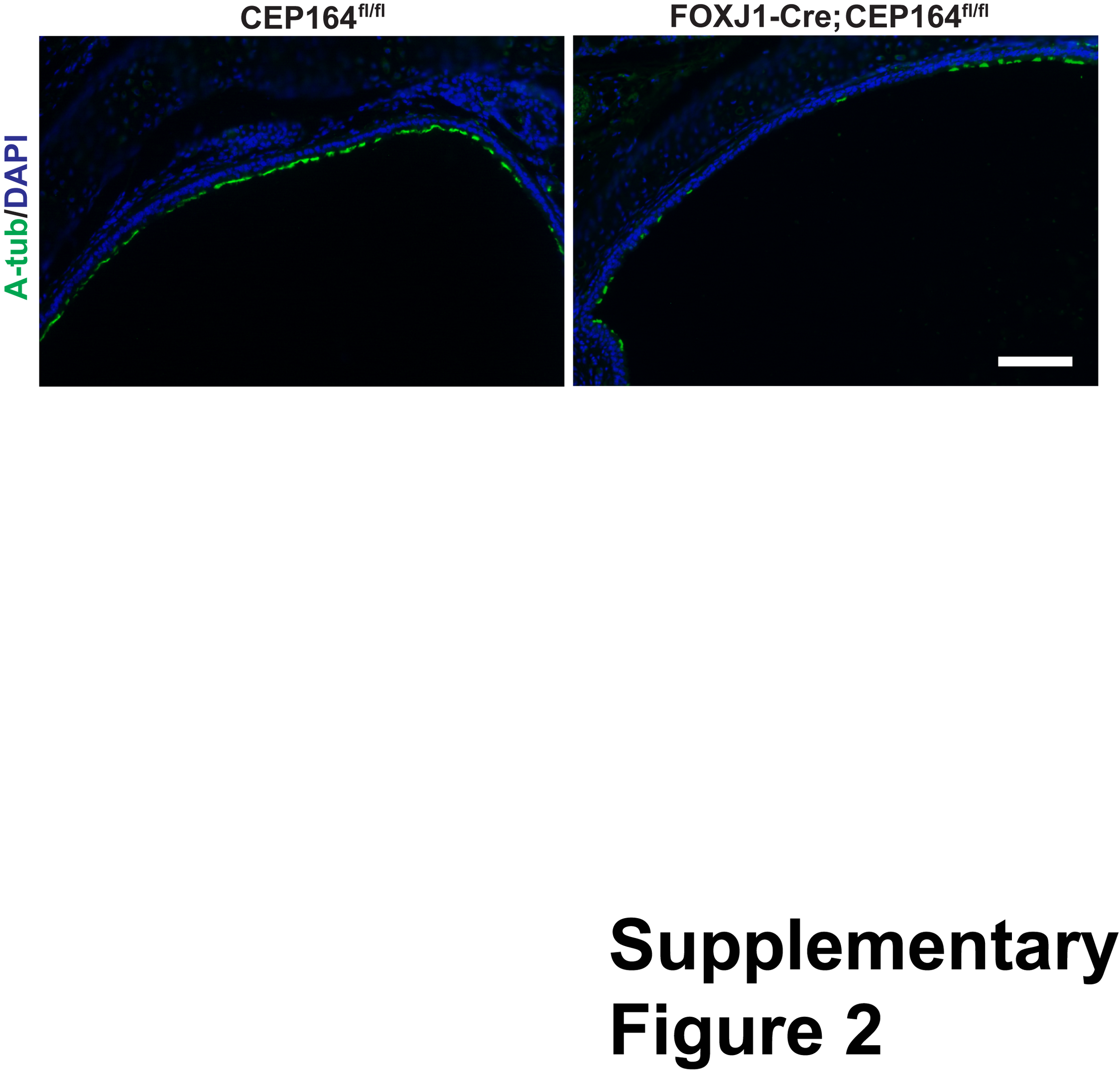

Supplement: S2 Fig — Tracheal sections from CEP164fl/fl and FOXJ1-Cre;CEP164fl/fl adult mice were immunostained for A-tub (green). Nuclei were detected with DAPI. Scale bar, 100 μm. (TIF) [file pgen.1007128.s002.tif]

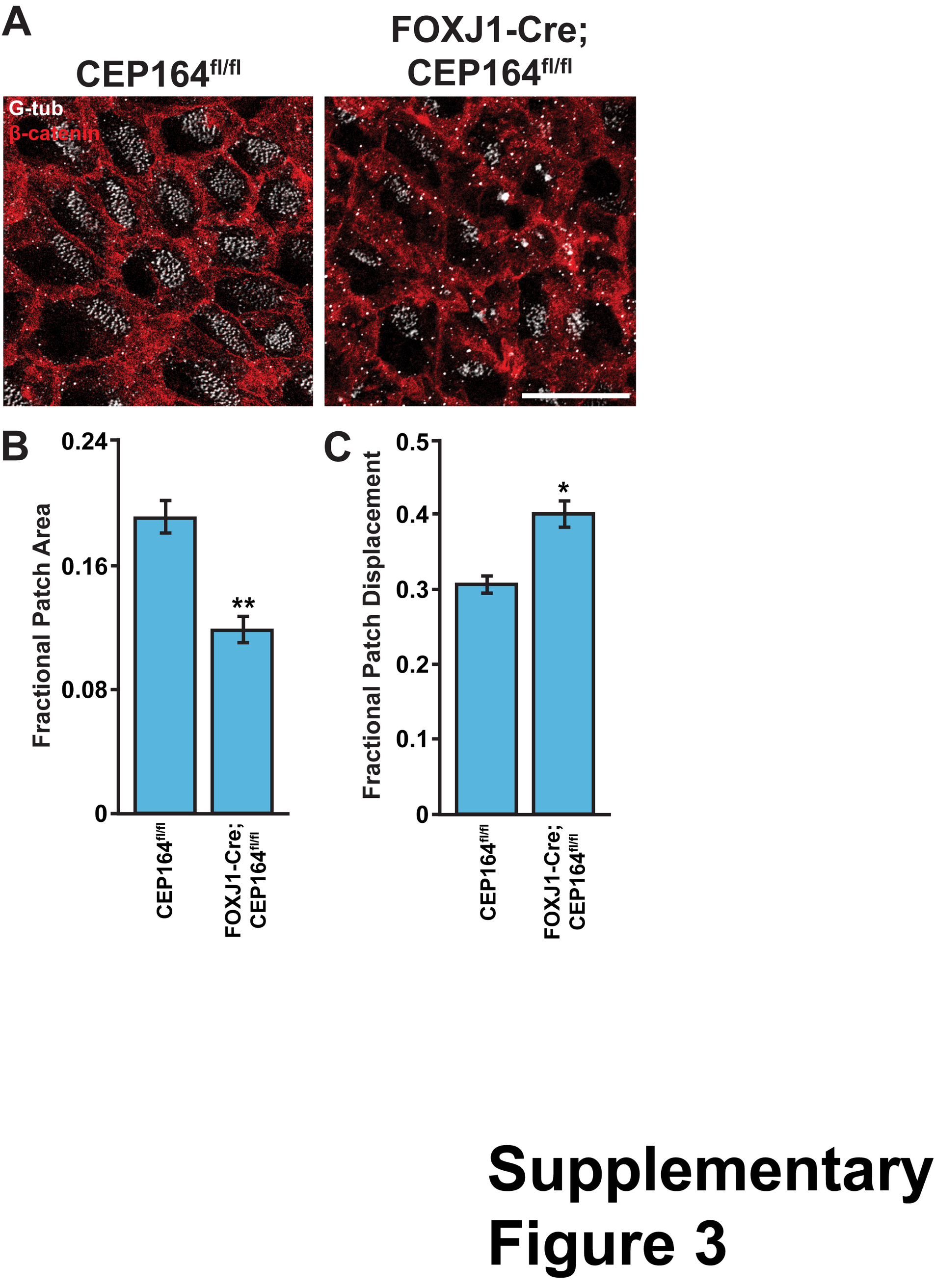

Supplement: S3 Fig — (A) SVZ whole mount preparations from CEP164fl/fl or FOXJ1-Cre;CEP164fl/fl adult mice were immunostained for G-tub (white) and β-catenin (red). β-Catenin demarcates the cell boundaries, and γ-tubulin labels basal bodies that are found in patches in ependymal multiciliated cells. Scale bar, 25 μm. (B) Quantification of basal body patch areas. Basal body patch areas relative to total apical cell surface areas are significantly reduced in CEP164-KO ependymal multiciliated cells. (C) Quantification of displacement of basal body patches. The displacement of the basal body patches from the cell center relative to the radius of the apical cell surface is significantly increased in the absence of CEP164. For all quantification, n = 3. Error bars represent ±SEM. *, p<0.05; **, p<0.01. (TIF) [file pgen.1007128.s003.tif]

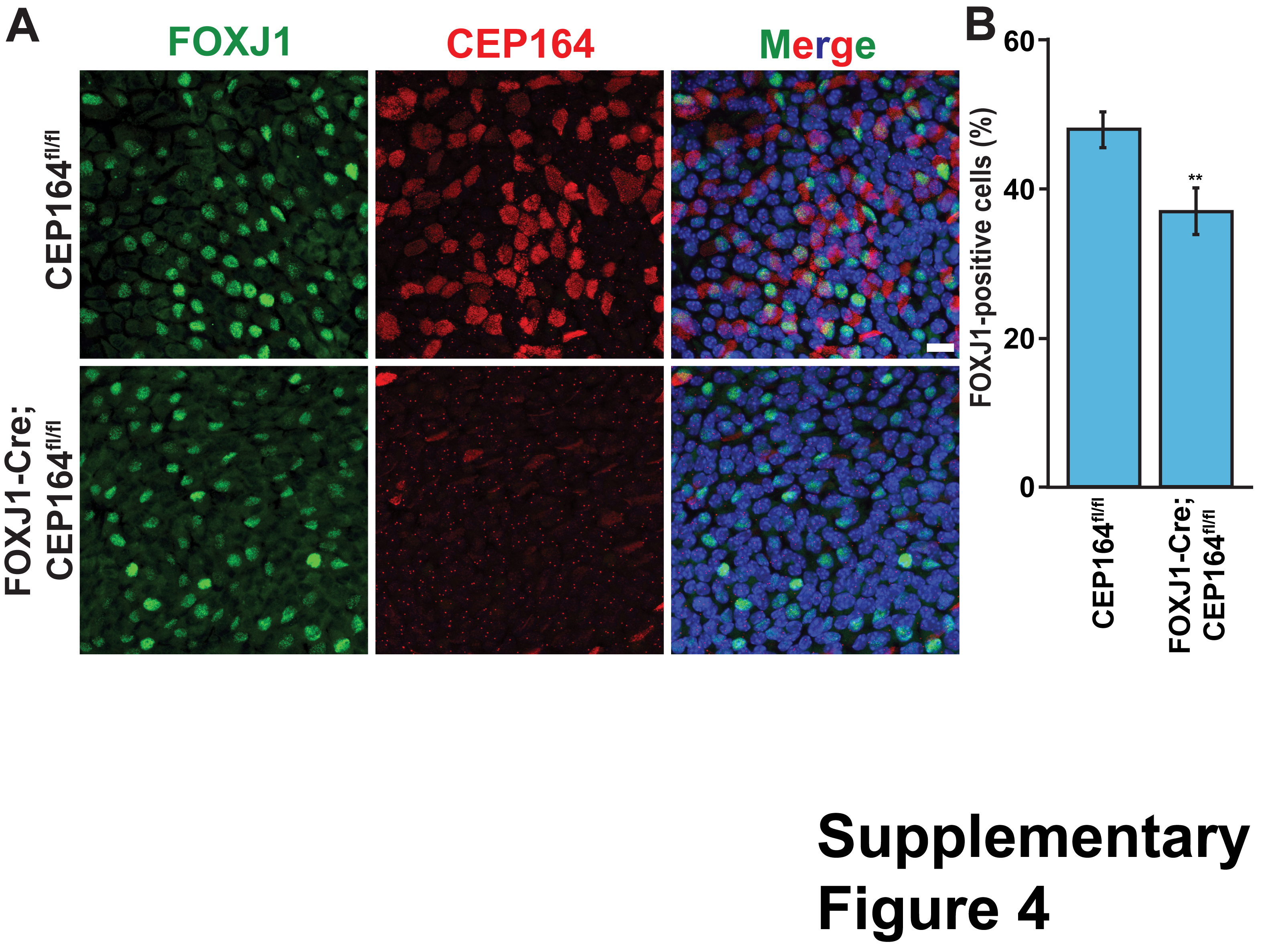

Supplement: S4 Fig — (A) MTECs were prepared from CEP164fl/fl and FOXJ1-Cre;CEP164fl/fl mice, fixed at ALId14, and immunostained for FOXJ1 (green) and CEP164 (red). Nuclei were stained using DAPI (blue). ~90% of multiciliated cells in MTEC cultures from FOXJ1-Cre;CEP164fl/fl mice lost CEP164 expression. Scale bar, 25 μm. (B) Quantification of FOXJ1-positive multiciliated cells. The percentage of FOXJ1-positive cells in FOXJ1-Cre;CEP164fl/fl MTECs was moderately reduced (~10%) in comparison to CEP164fl/fl MTECs. >500 cells were counted for each of three independent MTEC preparations per genotype. Error bars represent ±SEM. **, p<0.01. (TIF) [file pgen.1007128.s004.tif]

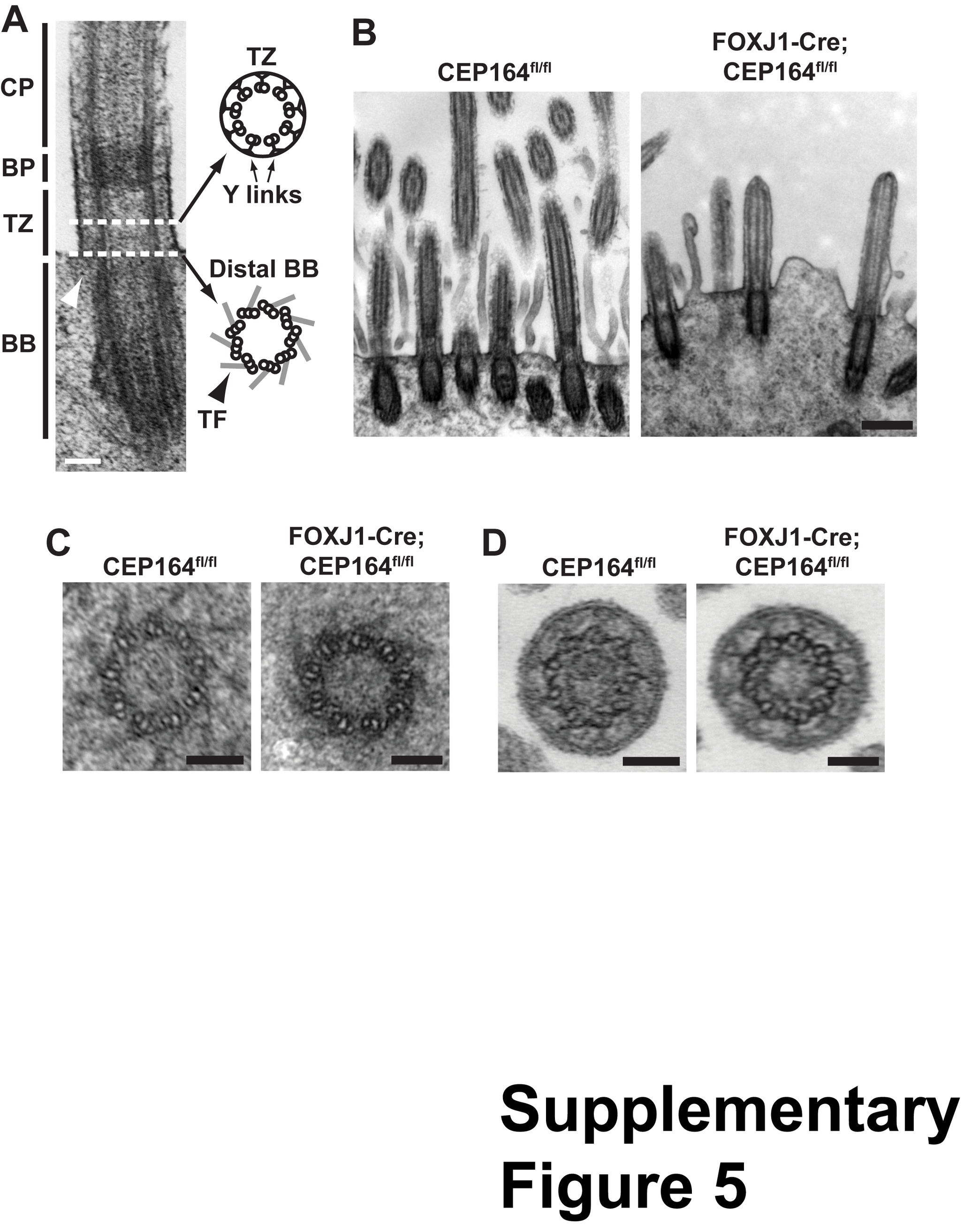

Supplement: S5 Fig — (A) Structure of multicilia. CP, cilia proper; BP, basal plate; TZ, transition zone, BB, basal body; TF, transition fiber (arrowheads). Scale bar, 100 nm. (B) Elongated cilia were abundant in cross-sections of tracheas from CEP164fl/fl adult mice while short cilia were frequently found in tracheas from FOXJ1;CEP164fl/fl adult mice. Scale bar, 500 nm. (C) Nine transition fibers from the microtubule triplets of the basal body were present in cross-sections of multicilia in ALId14 MTEC cultures from both CEP164fl/fl and FOXJ1-Cre;CEP164fl/fl mice. Scale bars, 100 nm. (D) Y-linkers within the transition zone were visible in cross-sections of multicilia in ALId14 MTEC cultures from both CEP164fl/fl and FOXJ1-Cre;CEP164fl/fl mice. Scale bars, 100 nm. (TIF) [file pgen.1007128.s005.tif]

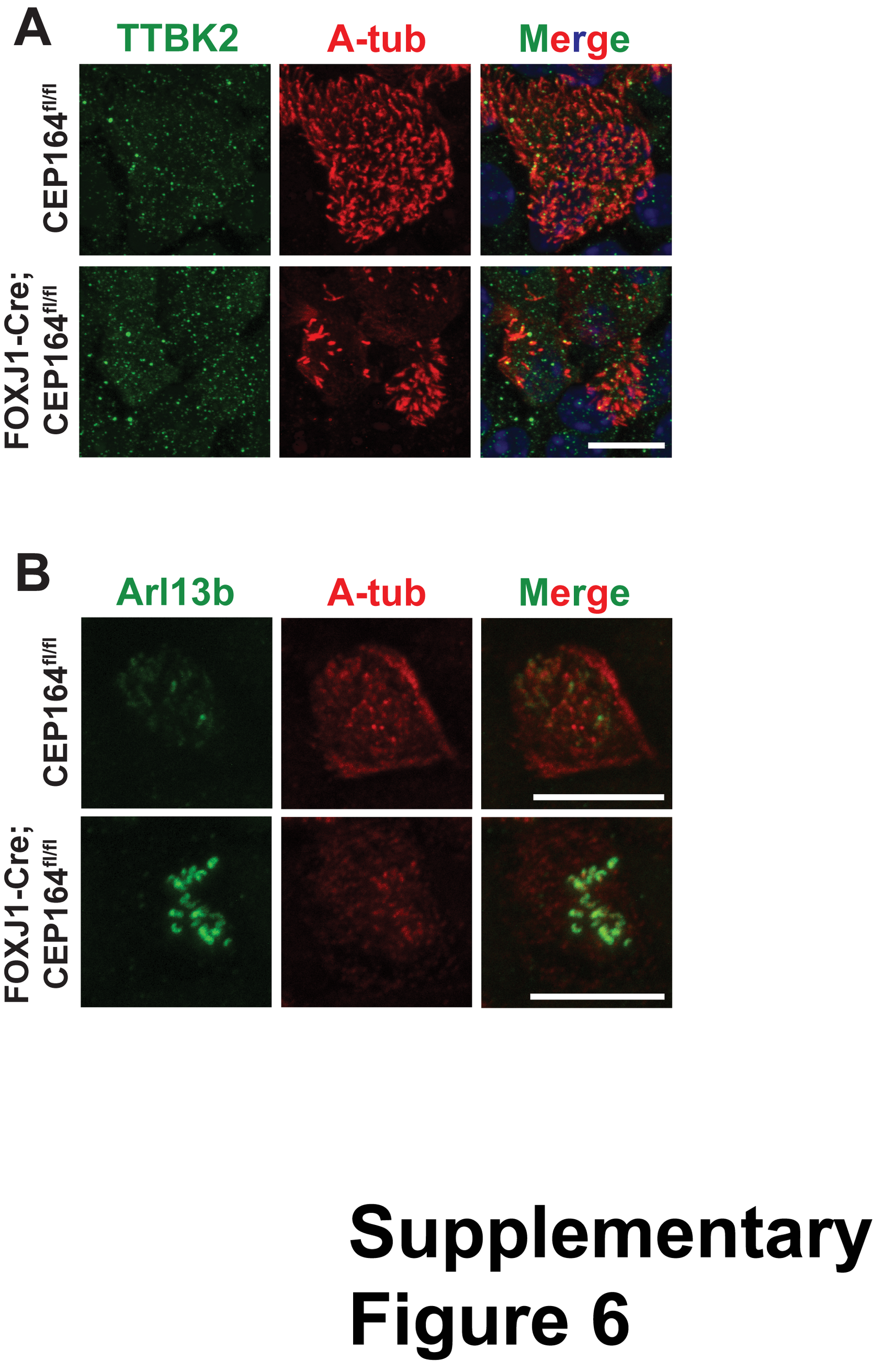

Supplement: S6 Fig — (A) ALId14 MTECs from CEP164fl/fl and FOXJ1-Cre;CEP164fl/fl mice were immunostained for TTBK2 (green) and the ciliary/basal body maker A-tub (red). Nuclei were detected with DAPI (blue). (B) ALId5 MTECs were immunostained for Arl13b (green) and A-tub (red) as indicated. Multiciliated cells at early ciliation phases are shown. Scale bars, 10 μm. (TIF) [file pgen.1007128.s006.tif]

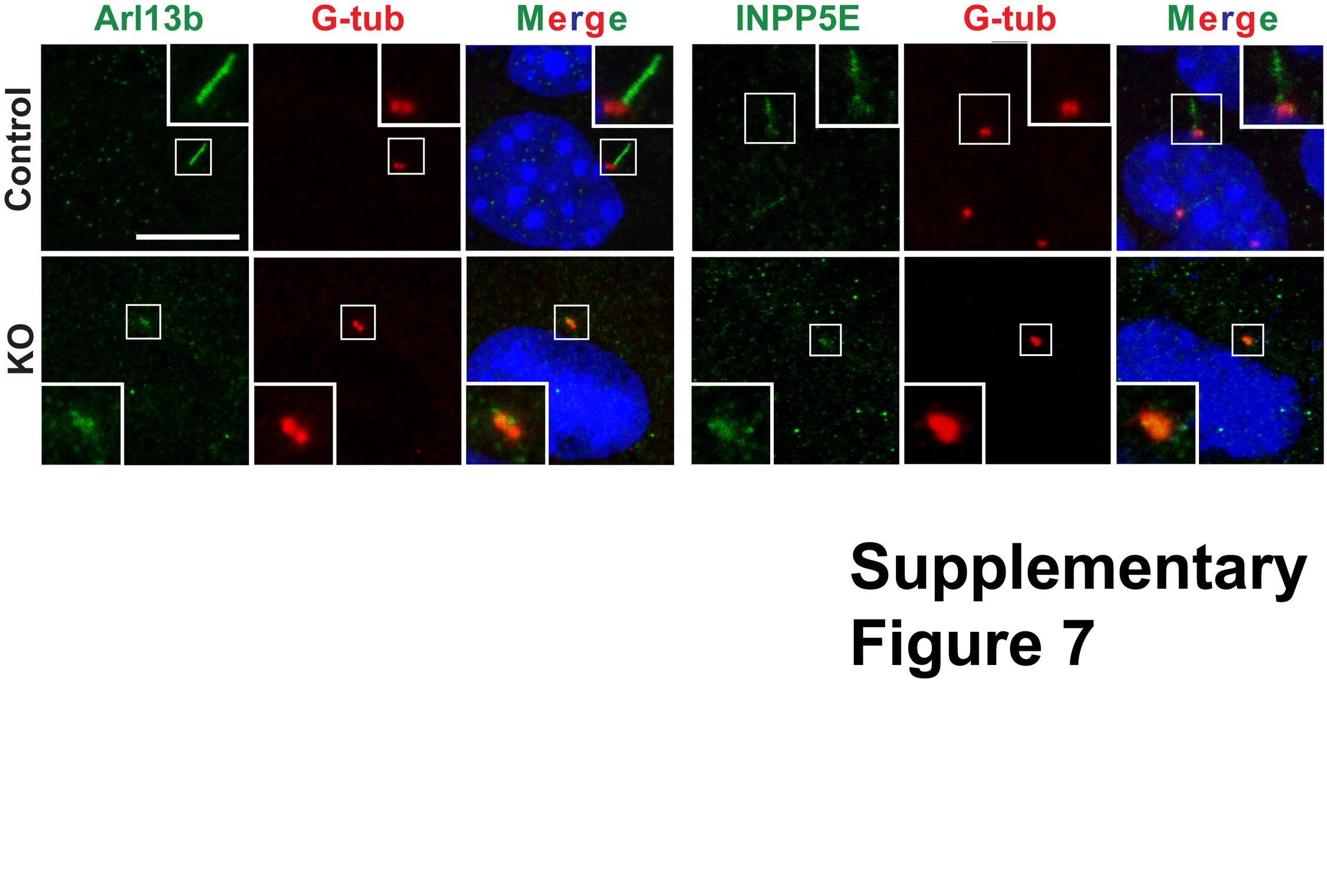

Supplement: S7 Fig — Mouse embryonic fibroblasts (MEFs) were prepared from E8.5 CEP164-KO or control embryos and serum-starved for 48 hours to induce primary cilia. MEFs were double-labeled for Arl13b or INPP5E (green) and the ciliary marker acetylated α-tubulin (A-tub). Nuclei were visualized by DAPI (blue). The boxed regions are enlarged in insets. Scale bar, 10 μm. (TIF) [file pgen.1007128.s007.tif]

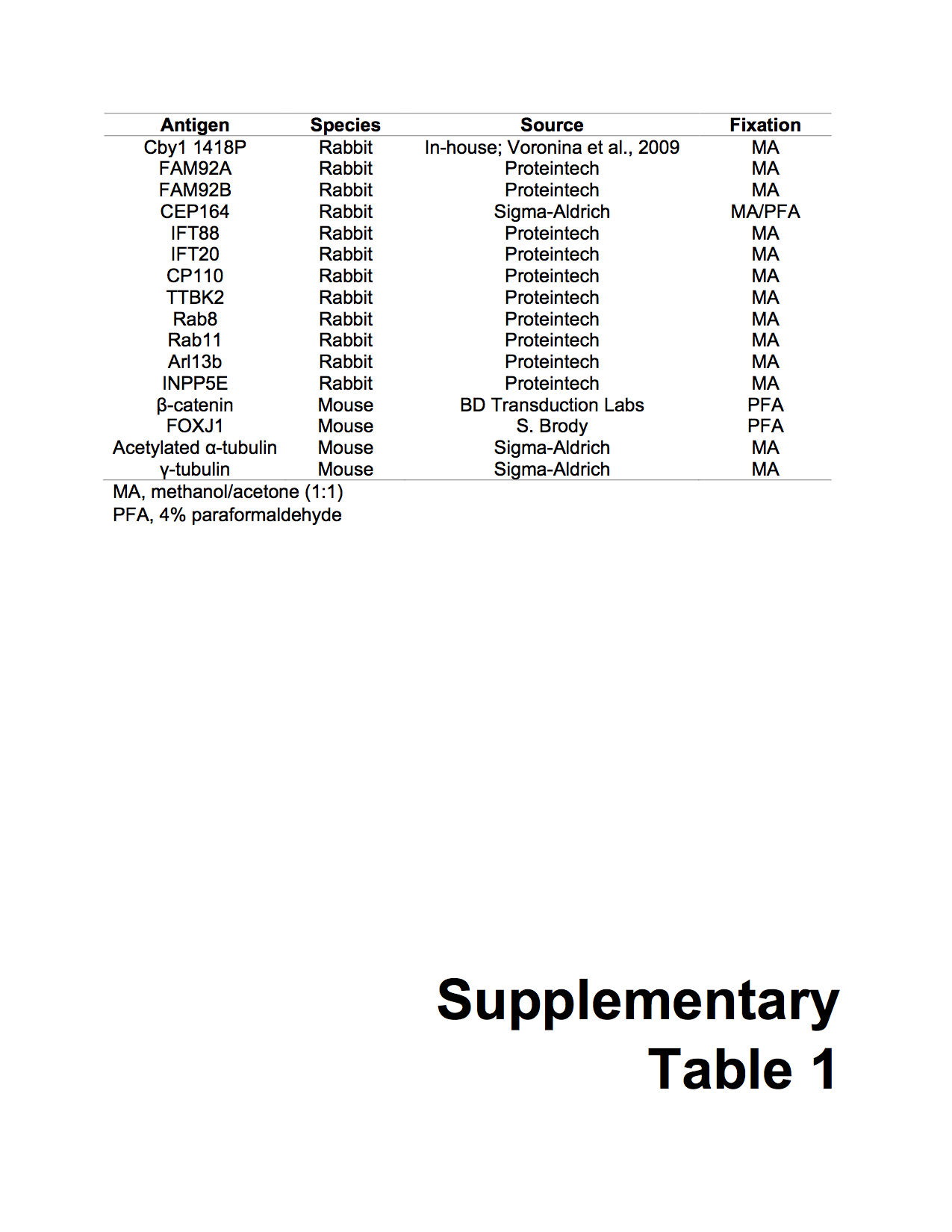

Supplement: S1 Table — (TIF) [file pgen.1007128.s008.tif]
